# Gene Expression Profiling of the Sciatic Nerve in Streptozotocin-Induced Diabetic Rats with Peripheral Neuropathy

**DOI:** 10.1155/2020/5283284

**Published:** 2020-05-20

**Authors:** Yiji Tu, Zenggan Chen, Feng Zhang, Zhenglin Di, Junhui Zhang, Li Cai

**Affiliations:** ^1^Department of Joint Surgery, Ningbo Sixth Hospital, Ningbo 315040, China; ^2^Department of Orthopedic Surgery, Zhongshan Hospital, Fudan University, Shanghai 200030, China; ^3^Joseph M. Still Burn and Reconstruction Center, Jackson, Mississippi 39201, USA; ^4^Department of Ophthalmology, Ningbo Eye Hospital, Ningbo 315040, China

## Abstract

**Aims:**

To investigate the candidate biomarkers and molecular mechanisms involved in the early phase of experimental diabetic peripheral neuropathy (DPN).

**Methods:**

Diabetes in Sprague-Dawley rats was induced with streptozotocin (STZ) treatment, followed with neurological tests and histological examinations to assess the neuropathic symptoms of DPN. Microarray was performed on the sciatic nerve tissues from control rats and DPN rats at then6th week after diabetes induction, and differentially expressed genes (DEGs) between them were identified and applied for further bioinformatic analyses.

**Results:**

Experimental DPN rats were successfully constructed, presenting significantly decreased withdrawal threshold and motor nerve conduction velocity, and typical histological changes in the sciatic nerve. 597 DEGs (186 up- and 411 downregulated) were identified in DPN rats. DEGs from the 3 most highly connected clusters in the protein-protein interaction network were enriched for biological processes or pathways such as “cell division,” “cell cycle,” “protein phosphorylation,” “chemokine signaling pathway,” “neuropeptide signaling pathway,” “response to drug,” “cellular response to insulin stimulus,” “PPAR signaling pathway,” and “glycerophospholipid metabolism.” Thirteen genes were identified as the hub DEGs in the PPI network. Eleven transcriptional factors (TFs) targeting 9 of the 13 hub DEGs were predicted.

**Conclusions:**

The present study identified a pool of candidate biomarkers such as *Cdk1*, *C3*, *Mapk12*, *Agt*, *Adipoq*, *Cxcl2*, and *Mmp9* and molecular mechanisms which may be involved in the early phase of experimental DPN. The findings provide clues for exploring new strategies for the early diagnosis and treatment of DPN.

## 1. Introduction

Diabetic neuropathy, the most common cause of neuropathy in developed countries, is estimated to affect up to 50% of patients with both type 1 and type 2 diabetes; nevertheless, it is often underdiagnosed and inadequately treated due to its unnoticeable and nonspecific manifestations during its early stages [1]. The most common form of diabetic neuropathy is chronic, distal, and symmetric sensorimotor polyneuropathy, also known as diabetic peripheral neuropathy (DPN). Other less common forms include asymmetric or focal neuropathies such as diabetic amyotrophy, cranial mononeuropathies, truncal radiculopathy, and pressure palsies [2, 3].

Gradual loss of protective sensation in distal extremities is the primary cause of foot ulceration and amputation in patients with DPN. Painful DPN develops in about 20% of diabetes, which can be very distressing and usually leads to sleep deprivation and depression [4]. At the advanced stages of DPN, motor nerve dysfunctions such as wasting of small muscles and limb weakness ensue. With regard to the clinical management of DPN, intensive blood glucose control may prevent its progression, while has little effect on the relief of neuropathic symptoms. American Diabetes Association (ADA) has suggested Duloxetine and Pregabalin as the 1^st^ line drugs and tricyclic compounds (TCAs) and Gabapentin as the 2^nd^ line drugs for the symptomatic relief in DPN patients. Unfortunately, their effects are often less than satisfactory and complicated by adverse events [5, 6].

The pathogenesis of DPN is multifactorial. Hyperglycemia is fundamentally linked to the onset and progression of DPN, regardless of the type of diabetes [7]. Other contributing factors include dyslipidemia, poor glycemic control, advanced age, smoking, heavy alcohol intake, hypertension, long duration of diabetes, and genetic factors [8]. Despite those extensively investigated etiologies, the specific molecular mechanisms contributing to DPN are not completely understood. Over the last decades, the paradigm for mechanism research has shifted from focusing on a single pathway to analyzing the entire biological system. More and more experimental and clinical studies investigating the transcriptional changes in the peripheral nerves of subjects with DPN have been carried out and revealed some promising therapeutic targets for DPN treatment [9–12].

The aim of our current study was to investigate the candidate biomarkers and molecular mechanisms involved in the early phase of experimental DPN via microarray analysis. Firstly, diabetes in rats was induced with streptozotocin (STZ) injection; then neurological tests were performed to assess the neuropathic symptoms of DPN. Histological examinations and microarray were performed on the sciatic nerve tissues from control and DPN rats at the 6th week after diabetes induction as we considered that the pathological changes that occurred at this time-point might better represent the early phase of DPN. Differentially expressed genes (DEGs) between control and DPN rats were identified and applied for further bioinformatic analyses, including the enrichment of biological processes or pathways, identification of hub DEGs, and prediction of potential transcriptional factors (TFs).

## 2. Materials and Methods

### 2.1. Animals and Groups

**Figure 1:**
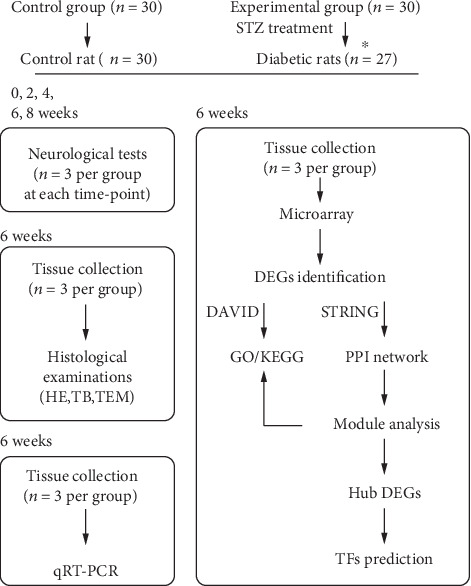
Study workflow. ^∗^After STZ treatment, 27 out of the 30 rats in experimental group were verified as diabetes and the remaining 3 rats were excluded from the study. Note that no rats were dead due to diabetes or anesthesia during the course of study. After neurological tests, rats were sacrificed without collecting the sciatic nerve tissues for the following experiments. Besides, total RNA isolated for microarray analysis was not used for qRT-PCR.

All animal experiments were carried out with the permission of the Animal Care Committee of Fudan University. Sixty adult male Sprague-Dawley rats (initial weight 250-300 g, from the Department of Laboratory Animal Science of Fudan University) were randomly assigned into two groups: control group (*n* = 30) and experimental group (*n* = 30). Diabetes in experimental group was induced with a single intraperitoneal injection of STZ (55 mg/kg). Rats in the control group were performed with a single intraperitoneal injection of 0.9% saline solution. Glucose level was evaluated using Sannuo strips on tail vein blood at day 3 after STZ injection and verified again at day 7 after STZ treatment. Only rats with blood glucose level ≥ 16.7 mmol/L were considered diabetic. The study workflow is shown in Figure 1.

### 2.2. Assessment of Neurological Deficits and Histological Changes

Pain threshold and motor nerve conduction velocity (MNCV) were measured at different time-points to assess the dynamics of neurological deficits during the course of diabetes. Pain threshold was measured in the right paw of rats with a set of von Frey filaments (North Coast Medical Inc., America), according to the methods described by Rojewska et al. [13]. After that, MNCV was measured in the right sciatic nerve of rats according to the protocols provided by O'Brien et al. [11].

Six weeks after the diabetes induction, 3 control rats and 3 diabetic rats were sacrificed and the right sciatic nerves (about 1 cm long) were harvested for histologic examination and morphometry analysis. Samples were fixed and cut into semithin (1 *μ*m) sections for hematoxylin-eosin (HE) staining and toluidine blue (TB) staining according to the provided protocols. Suitable areas from the semithin sections were chosen and cut into ultrathin (80 nm) sections for transmission electron microscopy according to the provided methods. Histological changes of the sciatic nerve were examined by optical microscopy (Olympus-DP71, Olympus, Japan) and transmission electron microscopy (Hitachi-7500, Hitachi, Japan).

### 2.3. Microarray Hybridization and Identification of DEGs

Six weeks after diabetes induction, 3 control rats and 3 diabetic rats were sacrificed and total RNA was extracted from the right sciatic nerves. Microarray hybridization was then performed according to the Agilent One-Color Microarray-Based Gene Expression Analysis protocol (Agilent Technologies). Pearson's correlation analysis was used to assess the correlation between samples by using R version 3.6.3 (https://www.r-project.org/). Quantile normalization and subsequent data processing were performed using the GeneSpring GX v12.1 software package (Agilent Technologies). The univariate *t*-test with a fold change ≥ 2 and *P* value < 0.001 were applied to identify the DEGs between control rats and diabetic rats.

### 2.4. Quantitative Real-Time Polymerase Chain Reaction (qRT-PCR)

Credibility of the microarray data was validated through qRT-PCR on 4 genes. Six weeks after diabetes induction, another 3 control rats and 3 diabetic rats were sacrificed and total RNA was extracted from the right sciatic nerves. Double-stranded cDNA was synthesized using a PrimeScript RT Reagent Kit (TaKaRa Biotechnology, Dalian, China) according to the manufacturer's instructions. LightCycler 480 SYBR Green I Master mix (Roche, Mannheim, Germany) was used for real-time PCR. *Gapdh* was used as the endogenous reference gene, and the 2^-*ΔΔ*CT^ method was used to quantify the relative expression level of each gene. The primer sequences, melting temperatures, and products sizes for selected genes are shown in Table 1.

### 2.5. Gene Ontology and Kyoto Encyclopedia of Genes and Genome Pathway Analyses

Functional annotation clustering of the DEGs was performed based on the Database for Annotation, Visualization, and Integrated Discovery (DAVID v6.8, https://david.abcc.ncifcrf.gov/). Biological process (BP), cellular component (CC), and molecular function (MF) items were represented via Gene ontology (GO), and pathway items were represented via Kyoto Encyclopedia of Genes and Genomes (KEGG). Only the GO items and KEGG pathways with a Benjamini-corrected *P* < 0.05 were considered statistically significant.

### 2.6. Module Analysis, Hub DEGs, and Master TFs

Protein-protein interaction (PPI) network for the up- and downregulated DEGs was constructed using the search tool for the retrieval of interacting gene/protein (STRING, v11.0, http://string.embl.de/) online database with the minimum required interaction score > 0.700 (high confidence). Cytoscape (v3.7.2, http://www.cytoscape.org/), an open source bioinformatics software platform, was used to visualize the PPI networks. In the network, the node represents a protein, and the line between nodes represents the interaction between proteins. The disconnected nodes in the network were hidden.

Module analysis on the PPI network was performed using the molecular complex detection (MCODE) plugin in Cytoscape under the parameters which include loops: false, degree cutoff: 2, node score cutoff: 0.2, haircut: true, fluff: false, K-Core: 2, and max. depth from seed: 100. In addition, the module analysis results were further analyzed for GO (BP) and KEGG pathway enrichment, and a Benjamini-corrected *P* < 0.05 was considered statistically significant. The CytoHubba plugin in Cytoscape was used to calculate the connectivity degree of each node in the PPI network. Nodes with higher connectivity degree tend to be more essential in maintaining the stability of the entire network. Hub genes were identified based on the connectivity degree of nodes in the PPI network.

The master TFs of hub DEGs were predicted using the iRegulon plugin in Cytoscape. By using this plugin, information from multiple TF databases such as Transfac, Jaspar, and Homer was retrieved and TF-binding motif enrichment analysis was performed to predict the TFs of a certain gene. The criteria for motif enrichment analysis were set as identity between orthologous genes ≥ 0.05, FDR on motif similarity ≤ 0.001, and normalized enrichment score (NES) > 5.

### 2.7. Statistical Analysis

Statistical analyses were performed using GraphPad Prism Software (version 8.2.1). All data were presented as the mean ± SD. The groups were analyzed using one-way or two-way analysis of variance (ANOVA), and individual groups were compared with Student's *t*-test. The criterion for statistical significance was *P* < 0.05.

## 3. Results

### 3.1. Neurological Tests and Histological Examinations

**Figure 2:**
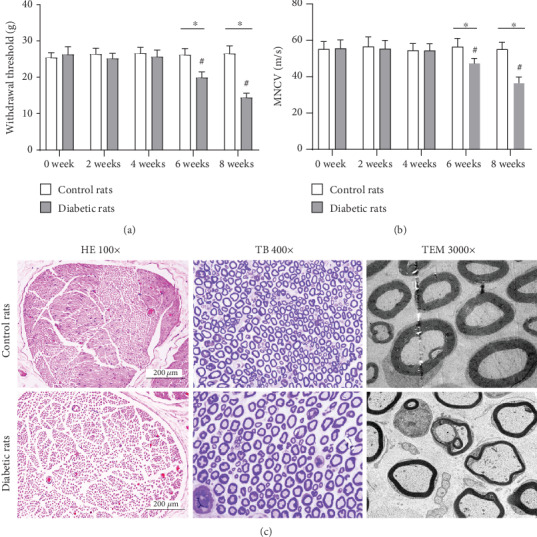
Neurological deficits and histological changes in the sciatic nerve of DPN and control rats. Compared with control rats, significantly lower withdrawal threshold and MNCV were observed in the sciatic nerve of diabetic rats since the 6th week after diabetes induction (a, b). ^∗^*P* < 0.05 vs. control rats; ^#^*P* < 0.05 vs. previous time point. Histological examination showed normal morphology of myelinated nerve fibers in the sciatic nerve of control rats. While typical histological changes of DPN including loss of myelinated fibers, thin myelin and breakdown of myelin were observed in the sciatic nerve of diabetic rats (c).

There were no significant differences in the withdrawal threshold and MNCV between control and diabetic rats within 6 weeks since diabetes induction, while since the 6th week after diabetes induction, the withdrawal threshold of diabetic rats (19.87 ± 1.64 g) was significantly lower than that of control rats (26.13 ± 1.78 g) (Figure 2(a)). Consistently, the MNCV of diabetic rats (47.35 ± 2.74 m/s) was significantly lower than that of control rats (56.53 ± 4.52 m/s) 6 weeks after diabetes induction (Figure 2(b)).

Under a light microscope, HE and TB staining of the sciatic nerve from control rats presented normal morphology of myelinated nerve fibers. However, loosely arranged nerve fibers and loss of myelinated nerve fibers were observed in the sciatic nerve of diabetic rats. Under a transmission electron microscope, numerous myelinated fibers with disproportionately thin, split, or ballooned myelin sheaths were observed in the sciatic nerve of diabetic rats (Figure 2(c)).

### 3.2. DEGs and Validation through qRT-PCR

**Figure 3:**
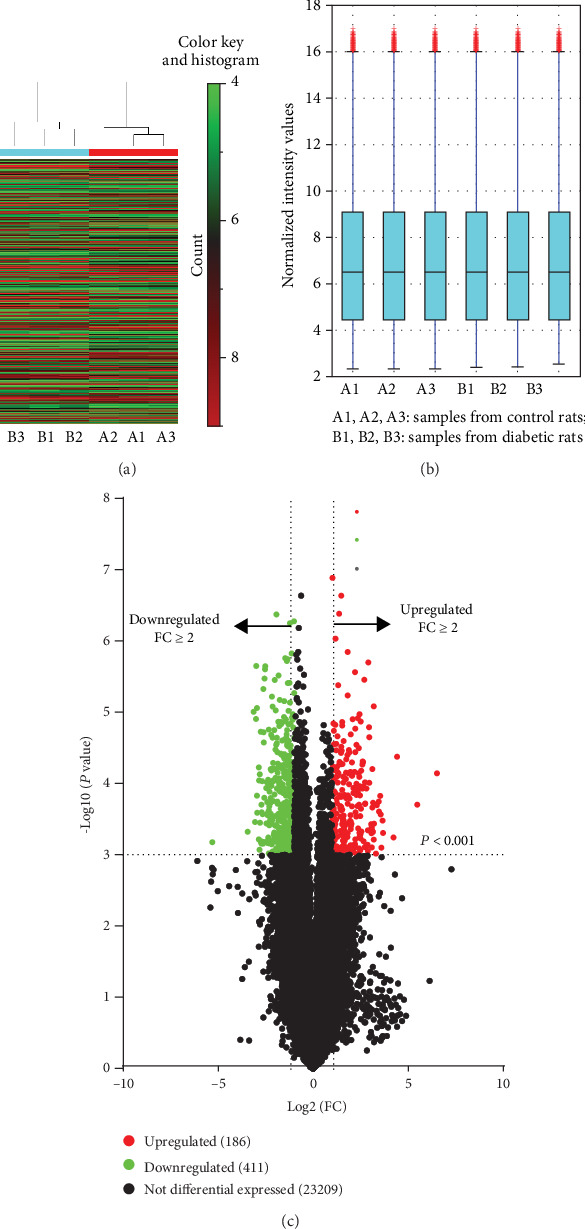
Heat map, box plot, and volcano plot of detected genes. 23806 gene IDs and their expression values were obtained after probe ID transformation and expression summarization. Heat map of detected genes from all samples (a). Red indicates higher gene expression, and green indicates lower gene expression. The box plot of the intensity of all samples demonstrated that the expression values of each sample were close to the same after normalization (b). The univariate *t*-test with a fold change > 2 and *P* < 0.001 was applied to identify the DEGs between control rats and diabetic rats. 186 upregulated genes (red spots) and 411 downregulated genes (green spots) were identified as the DEGs between groups (c).

Correlation plot showed strong correlation between samples within each group (Supplemental Figure 1). 23806 gene IDs and their expression values were obtained after probe ID transformation and expression summarization.  Figure 3(a) shows the heat map of detected genes from all samples. The box plot of the intensity of all samples demonstrated that the expression values of each sample were close to the same after normalization (Figure 3(b)). After gene screening (fold change ≥ 2 and *P* < 0.001), a total of 597 DEGs (186 up- and 411 downregulated genes) were identified between groups (Figure 3(c)). Supplemental Table 1 shows the complete list of DEGs.  Table 2 shows the top 10 up- and downregulated DEGs based on fold change, respectively.

**Figure 4:**
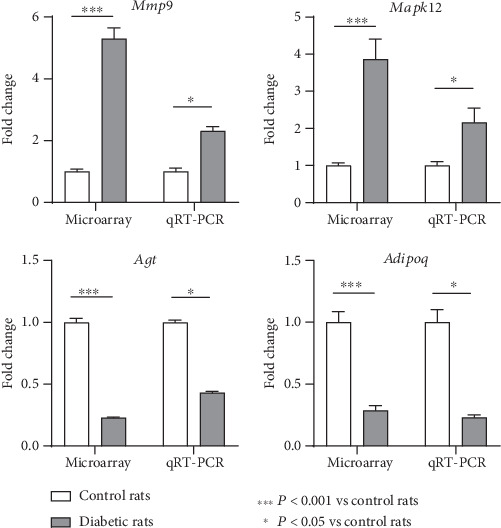
Validation of the microarray data through qRT-PCR. Four specific genes of interest including 2 upregulated (*Mmp9* and *Mapk12*) and 2 downregulated (*Agt* and *Adipoq*) genes were selected for qRT-PCR validation. The qRT-PCR results showed that, compared with control rats, the gene expression levels of *Mmp9* and *Mapk12* were significantly increased in diabetic rats, with a fold change of 2.31 and 2.16, respectively. With regard to *Agt* and *Adipoq*, their expression levels were significantly decreased in diabetic rats compared with control rats, with a fold change of 0.43 and 0.23, respectively. ^∗∗∗^*P* < 0.001 vs. control rats; *P* < 0.05 vs. control rats. The expression level changes of these 4 genes validated the credibility of the microarray data.

Four specific genes including 2 upregulated genes (*Mmp9* and *Mapk12*) and 2 downregulated genes (*Agt* and *Adipoq*) were selected for qRT-PCR validation in consideration of their potentially important roles in the pathogenesis of experimental DPN. Compared with control rats, the gene expression levels of *Mmp9* and *Mapk12* were significantly increased in diabetic rats, with a fold change of 2.31 and 2.16, respectively. The gene expression levels of *Agt* and *Adipoq* were significant decreased in diabetic rats compared with control rats, with a fold change of 0.43 and 0.23, respectively. The qRT-PCR results reflected comparable profiles, validating the credibility of the microarray data (Figure 4). Our raw expression data have been submitted to the GEO database and can be obtained via the accession number GSE147732.

### 3.3. GO Items and KEGG Pathways

**Figure 5:**
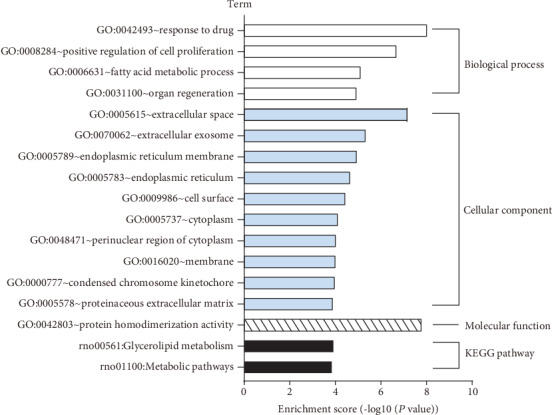
GO and KEGG pathway enrichment analyses based on the 597 DEGs. GO and KEGG pathway enrichment analyses demonstrated that 4 BP items (“response to drug,” “positive regulation of cell proliferation,” “fatty acid metabolic process,” and “organ regeneration”), 10 CC items (“extracellular space,” “extracellular exosome,” “endoplasmic reticulum membrane,” “endoplasmic reticulum,” “cell surface,” “cytoplasm,” “perinuclear region of cytoplasm,” “membrane,” “condensed chromosome kinetochore,” and “proteinaceous extracellular matrix”), 1 MF item (“protein homodimerization activity”), and 2 KEGG items (“glycerolipid metabolism” and “metabolic pathways”) were enriched for the combined 597 DEGs.

The 186 up- and 411 downregulated DEGs were combined for functional annotation clustering based on the DAVID online tool. As a result, 4 BP items (“response to drug,” “positive regulation of cell proliferation,” “fatty acid metabolic process,” and “organ regeneration”), 10 CC items (“extracellular space,” “extracellular exosome,” “endoplasmic reticulum membrane,” “endoplasmic reticulum,” “cell surface,” “cytoplasm,” “perinuclear region of cytoplasm,” “membrane,” “condensed chromosome kinetochore,” and “proteinaceous extracellular matrix”), 1 MF item (“protein homodimerization activity”), and 2 KEGG items (“glycerolipid metabolism” and “metabolic pathways”) were presented (Figure 5).

### 3.4. Module Analysis, Identification of Hub DEGs, and Prediction of TFs

**Figure 6:**
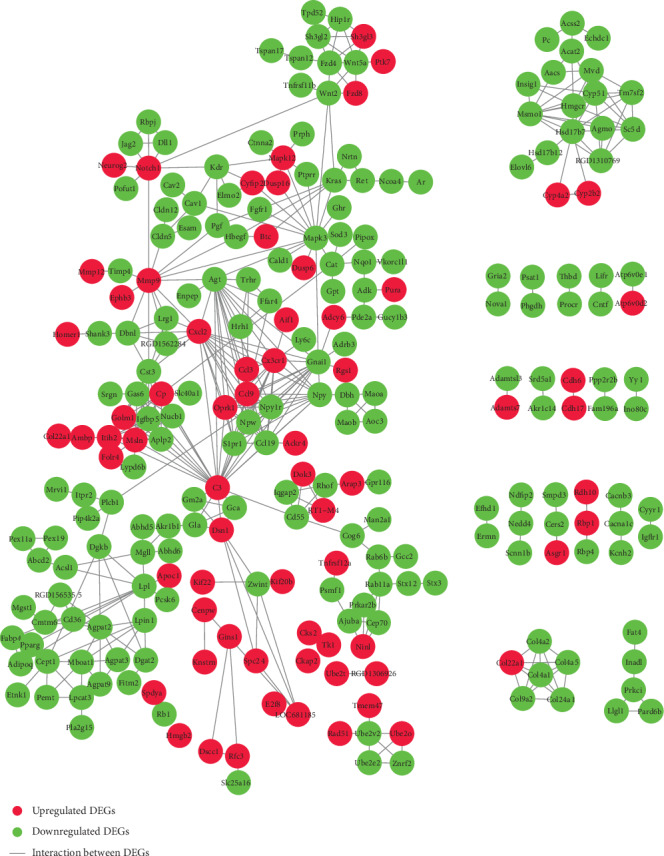
PPI network based on the 597 DEGs. The PPI network for the DEGs was constructed using the STRING with the minimum required interaction score > 0.700 (high confidence) and then visualized using the Cytoscape. A total of 248 nodes (in red color) and 783 edges were yielded in the PPI network for those up- (red nodes) and downregulated (green nodes) DEGs. Note that the disconnected nodes (DEGs) in the network were hidden.

**Figure 7:**
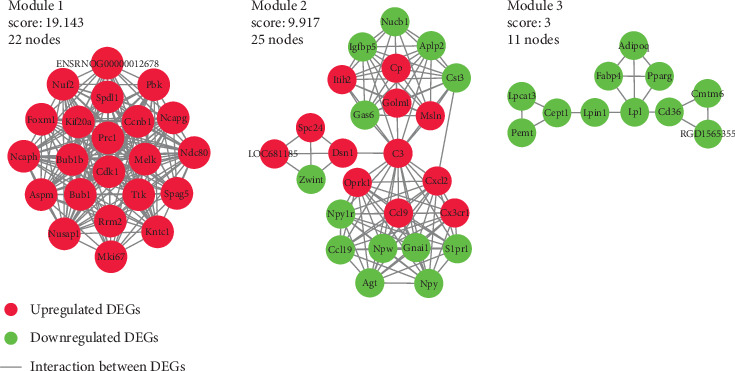
Three most highly connected clusters in the PPI network. With the criteria of minimum node number > 10 and cluster score ≥ 3, 3 most highly connected clusters were extracted from the PPI network by MCODE plugin in Cytoscape. DEGs in module 1 were all upregulated while in module 3 they were all downregulated.

PPI networks for the 597 DEGs were constructed using the STRING and visualized using the Cytoscape. A total of 248 nodes and 783 edges were presented in the PPI network based on those up- and downregulated DEGs (Figure 6). With the criteria of minimum node number > 10 and cluster score ≥ 3, 3 most highly connected clusters were extracted from the PPI network through MCODE analysis (Figure 7). DEGs in module 1 were all upregulated while those in module 3 were all downregulated. Further module analysis demonstrated that DEGs from the 3 modules were enriched in GO (BP) items or KEGG pathways including “cell division,” “cell cycle,” “protein phosphorylation,” “chemokine signaling pathway,” “neuropeptide signaling pathway,” “response to drug,” “cellular response to insulin stimulus,” “PPAR signaling pathway,” and “glycerophospholipid metabolism (Table 3).

**Figure 8:**
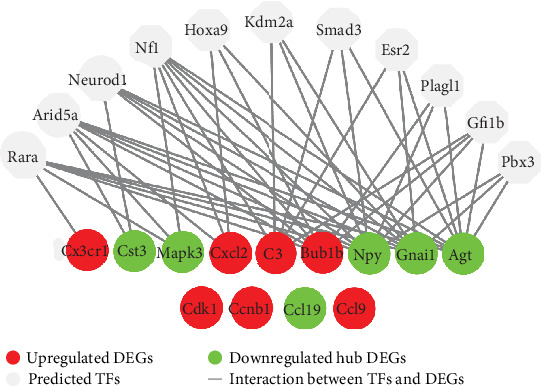
Prediction of TFs for hub DEGs. The master TFs of hub DEGs were predicted using the iRegulon plugin in Cytoscape. The criteria for motif enrichment analysis were set as identity between orthologous genes ≥ 0.05, FDR on motif similarity ≤ 0.001, and normalized enrichment score (NES) > 5. As shown, a total of 11 TFs (Rara, Arid5a, Neurod1, Nf1, Hoxa9, Kdm2a, Smad3, Esr2, Plagl1, Gfi1b, and Pbx3) were predicted to regulate 9 of the 13 hub DEGs.

CytoHubba plugin in Cytoscape was used to identify the hub DEGs of the PPI network. As DEGs in module 1 were highly connected with each other, they produced relatively higher connectivity degree compared with other DEGs of the PPI network. In order to diminish the imbalance, 3 and 10 hub DEGs were identified from module 1 and the remaining DEGs, respectively, to represent the overall hub DEGs of the whole PPI network (Table 4). Among them, *Cdk1*, *Bub1b*, *Ccnb1*, *C3*, *Cxcl2*, *Ccl9*, *Cx3cr1*, and *Mmp9* were upregulated, while *Agt*, *Gnai1*, *Mapk3*, *Cst3*, and *Npy* were downregulated. A total of 11 TFs (Rara, Arid5a, Neurod1, Nf1, Hoxa9, Kdm2a, Smad3, Esr2, Plagl1, Gfi1b, and Pbx3) were predicted to regulate 9 of the 13 hub DEGs using the iRegulon plugin in Cytoscape with an NES > 5 (Table 5, Figure 8).

## 4. Discussion

Three (*Cdk1*, *Bub1b*, and *Ccnb1*) of the 13 hub DEGs were identified from module 1 of the PPI network, and they were all upregulated. Functional enrichment analyses indicated that they were associated with “cell division,” “protein phosphorylation,” and “cell cycle.” This is in accordance with the study by Gu et al., in which a number of DEGs identified from the sciatic nerve of type 1 diabetic mice were enriched in the BP item “cell proliferation” [14]. They considered that the abnormal pain responses in neuropathic pain were associated with the proliferation of nerve cells including microglia and astrocyte. It was indicated that loss of C-fibers in type 1 diabetic mice was accompanied with increased frequencies of denervated Schwann cells and regenerating fibers [15]. Though we also observed abnormal pain response (significantly lower withdrawal threshold) in the DPN rats of our present study, further studies are needed to identify its underlying molecular mechanisms and its correlation with the expression levels of specific DEGs.

Ten hub DEGs (*C3*, *Agt*, *Cxcl2*, *Gnai1*, *Mapk3*, *Ccl9*, *Cst3*, *Cx3cr1*, *Mmp9*, and *Npy*) were identified from the remaining DEGs of the PPI network. Functional enrichment analyses demonstrated that several upregulated hub DEGs including *Cxcl2*, *Cx3cr1*, and *Ccl9* were associated with “chemokine signaling pathway.” Among them, *Cxcl2* represented one of the top 10 upregulated DEGs with a fold change of 21.200. Chemokine (C-X-C motif) ligand 2 (CXCL2) is a member of chemokines and plays an important role in inflammatory reactions. Evidence has indicated a central role for inflammatory mechanisms in the development of DPN [16].

*Mapk12* was upregulated with a fold change of 3.867 in microarray and further verified through qRT-PCR. Mitogen-activated protein kinase 12 (MAPK12), also known as p38*γ*, is one of the four isoforms (*α*, *β*, *γ*, and *δ*) of p38 kinases [17]. Increased p38/MAPK pathway activation by inflammatory stimuli has been widely reported in the peripheral nerve tissues of diabetic mice or rats with DPN. Cheng et al. reported that the persistent hind paw mechanical allodynia in STZ-induced diabetic rats was positively correlated with increased P-p38/MAPK activation in activated microglia [18]. Similarly, Feldman et al. found that nerve growth factor-induced mechanical allodynia in the *db/db* mouse was mediated by the phosphorylation of p38 and the upregulation of multiple inflammatory mediators in lumbar dorsal root ganglia [19]. Several studies have demonstrated that inhibitors targeting the p38/MAPK pathway ameliorated inflammatory processes and neurological deficits in experimental DPN [20–22]. While it has been indicated that the regulatory function of p38 kinases on inflammatory processes is mainly through p38*α* (and probably *β*), the role of p38*γ* and p38*δ* in inflammation is unknown [17].

*Mmp12* represented the most upregulated gene with a fold change of 90.673, and *Mmp9* was one of the upregulated hub DEGs with a fold change of 5.300. As known, matrix metalloproteinases (MMPs) are a family of proteolytic enzymes which play important roles in the structural development and maintenance of the nervous system. Larsen et al. found that both MMP9 and MMP12 were elevated during myelin formation in dissected mouse optic nerves, while mice deficient in MMP9 and MMP12 exhibited impaired myelin formation, suggesting their beneficial roles in myelinogenesis [23]. Interestingly, it was reported in some studies that elevated MMP9 contributed to the pathogenesis of neuropathic pain following nerve injury, chiefly through its involvement in neuroinflammation [24, 25]. Hinder et al. found that MMP12 was highly up-regulated in the sciatic nerve of *db/db* diabetic mice with DPN and suggested it may play a role in DPN progression [26]. Thus, further investigation is needed to fully understand the protective or destructive role of MMPs in DPN.

*Agt* was one of the downregulated hub DEGs with a fold change of 4.348. Angiotensinogen (AGT), encoded by *Agt*, is a member of the renin angiotensin aldosterone system, the activation of which has been suggested a vital mechanism in the pathogenesis of DPN [48]. In the presence of hyperglycemia, the tissue level of angiotensin II (Ang II) is increased, which causes the accumulation of NADP oxidase and enhances oxidative stress. Consequently, microvascular damage occurs in the peripheral nerves and initiates the development of DPN [27]. Evidence has suggested that AGT levels are increased in the intrarenal renin-angiotensin system in diabetes, and the enhanced intrarenal AGT levels may contribute to the progression of diabetic nephropathy [28, 29]. However, we failed to find any studies investigating the direct correlation between AGT levels in peripheral nerve tissue and DPN progression through our extensive literature search.

Several GO (BP) or KEGG pathway items related to fat or lipid metabolism, including “fatty acid oxidation,” “brown fat cell differentiation,” “fatty acid metabolic process,” “PPAR signaling pathway,” “AMPK signaling pathway,” and “adipocytokine signaling pathway,” were enriched based on those downregulated DEGs in module 3. Correlations between altered lipid metabolism and the increased incidence of neuropathy have been identified in both type 1 and type 2 diabetes [30, 31]. Zhou et al. screened the DEGs between progressive and nonprogressive DPN patients and proposed that the inhibited lipid metabolism of Schwann cells might be crucial in the pathogenesis of progressive DPN [11].

*Adipoq* was downregulated with a fold change of 4.348 in microarray and further verified through qRT-PCR. Adiponectin, encoded by *Adipoq*, is secreted mainly by the adipose tissue, while it is also expressed in other tissues including osteoblasts, liver parenchymal cells, myocytes, and epithelial cells. Adiponectin improves insulin sensitivity and exhibits antidiabetic, anti-inflammatory, and antiatherogenic effects [32]. It is generally accepted that serum levels of adiponectin are inversely correlated to obesity/type 2 diabetes [33]. However, the relationship between serum adiponectin levels and diabetes-related microvascular and peripheral nervous complications is controversial. Kato et al. and Matsuda et al. demonstrated that serum adiponectin levels did not correlate with DPN in type 2 diabetic patients [34, 35]. While Ji et al. showed in their study that serum levels of adiponectin were markedly reduced in type 2 diabetic patients with DPN compared with healthy normal controls and type 2 patients without DPN [36]. Our current study found a decreased expression level of *Adipoq* in the sciatic nerve of DPN rats, while further experiments are warranted to determine in what cells it is expressed and to monitor its dynamic changes during the development of DPN.

Currently, most of the gene expression profiling studies in animal models of DPN were reported by Feldman and her colleges [10, 11, 26, 37]. Either type 1 (STZ-induced) or type 2 (BTBR *ob/ob* mice or BKS *db/db*) diabetic mice were selected as the experimental models of DPN in their studies. Microarrays were performed on the sciatic nerve of their experimental mice and followed with further bioinformatic analyses. Several biological processes or pathways including “lipid metabolism,” “PPAR signaling,” and “inflammation and immune response” were found to be commonly dysregulated in the DPN mice with type 2 diabetes across their studies [10, 11, 26]. While in the DPN mice with type 1 diabetes, “metabolism,” “metal ion binding,” “mRNA processing,” “cellular regulation,” and “mitochondria and energy production” were the top 5 dysregulated functional clusters, as reported in one of their studies [37].

STZ-induced diabetic rats have been widely used for the histopathological, pharmaceutical, or neurological research of experimental DPN [38–41]. Yet few studies have performed microarray experiment on the peripheral nerve tissue of DPN rats with STZ-induced diabetes. Price et al. investigated the gene expression changes in the dorsal root ganglia of male Wistar rats with STZ-induced diabetes. Gene ontology demonstrated that genes involved in “glucose metabolism” and “synaptic vesicle” were significantly upregulated after 1 and 4 weeks of diabetes, while genes involved in “cholesterol metabolism” were significantly downregulated after 8 weeks of diabetes [42]. Of note, Freeman et al. recently reported that metabolic dysfunction was restricted to the sciatic nerve, but not the dorsal root ganglion and trigeminal ganglia, in the experimental DPN rats based on metabolomics and proteomics analyses [43]. In agreement with their standpoint, we consider that the peripheral nerve is the most affected organ in the peripheral nervous system during the pathogenesis of DPN. It is exciting to find that the sciatic nerves of our STZ-induced diabetic rats presented typical histological changes of DPN after 6 weeks of diabetes, including loss of myelinated fibers, thin myelin, and breakdown of myelin, and shared many biological disruptions or signaling pathway changes indicated in those previously published animal or human microarray studies of DPN [10–12, 26, 37, 44]. Nevertheless, it should be noted that the sciatic nerve includes a heterogeneous mix of Schwann cells, fibroblasts, adipocytes, vascular endothelial cells, and even circulating immune cells. Therefore, DEGs from the sciatic nerve (or other peripheral nerves) of DPN should not be simply attributed to the dysfunction or lesion of Schwann cells.

## 5. Conclusions

The present study identified a pool of candidate biomarkers such as *Cdk1*, *C3*, *Mapk12*, *Agt*, *Adipoq*, *Cxcl2*, and *Mmp9* and molecular mechanisms which may be involved in the early phase of experimental DPN. The findings provide clues for exploring new strategies for the early diagnosis and treatment of DPN.

## Figures and Tables

**Table 1:** Primers used in qRT-PCR validation.

Gene	Primer sequences	Products' length	*T* (°C)
Mmp9	F: 5′-ACCCTGCGTATTTCCATTCATC-3′	169	60
	R: 3′-CGAGTTGCCCCCAGTTACAGT-5′		60

Mapk12	F: 5′-CTTGAGGAATGGAAGCGTGTT-3′	274	60
	R: 3′-TAGTGCTTGGGAGGTAGAGG-5′		60

Agt	F: 5′-GGTTCTCAACAGCATCCTCCTT-3′	145	60
	R: 3′-ACCTGAGTCCCGCTCGTAGA-5′		60

Adipoq	F: 5′-TGTTCCTCTTAATCCTGCCCAG-3′	239	60
	R: 3′-TCCTGTCATTCCAGCATCTCCT-5′		60

Gapdh	F: 5′-TTCCTACCCCCAATGTATCCG-3′	281	60
	R: 3′-CATGAGGTCCACCACCCTGTT-5′		60

Four genes including 2 upregulated genes (*Mmp9* and *Mapk12*) and 2 downregulated genes (*Agt* and *Adipoq*) were selected for qRT-PCR validation. *Gapdh* was used as the endogenous reference gene.

**Table 2:** Top 10 up- and downregulated DEGs based on fold change.

Gene symbol	Genbank accession	Fold change	Regulation	*P* value
*Mmp12*	NM_053963	90.673	Up	0.0000
*Ucn2*	NM_133385	44.201	Up	0.0002
*Cxcl2*	NM_053647	21.200	Up	0.0000
*C3*	NM_016994	18.631	Up	0.0006
*Tagln3*	NM_031676	12.766	Up	0.0005
*Notch1*	NM_001105721	12.491	Up	0.0003
*Scn3b*	NM_139097	11.931	Up	0.0008
*Cthrc1*	NM_172333	11.618	Up	0.0003
*Atp6v0d2*	NM_001011972	11.413	Up	0.0001
*Eltd1*	AF192402	11.019	Up	0.0002
*Lrrn1*	NM_001037363	10.994	Down	0.0005
*Hapln2*	NM_022285	8.858	Down	0.0000
*Lpl*	NM_012598	8.382	Down	0.0003
*Cdo1*	NM_052809	8.091	Down	0.0000
*Dbh*	NM_013158	8.064	Down	0.0000
*Pmp2*	NM_001109514	7.860	Down	0.0001
*Lrp2*	NM_030827	7.805	Down	0.0000
*Col9a2*	NM_001108675	7.737	Down	0.0003
*Hist1h4b*	NM_022686	7.316	Down	0.0000
*Efhd1*	NM_001109310	7.291	Down	0.0006

As shown, *Mmp12* was the most upregulated gene with a fold change of 90.673 and *Lrrn1* was the most downregulated gene with a fold change of 10.994 among those identified DEGs.

**Table 3:** GO (BP) and KEGG pathway enrichment analyses of the 3 modules.

Term	Genes	*P* value
Module 1: GO (BP)		
Cell division	*Ccnb1*, *Cdk1*, *Ncaph*, *Spag5*, *Nuf2*, *Bub1b*, *Spdl1*	0.0000
Protein localization to kinetochore	*Cdk1*, *Bub1b*, *Ttk*, *Spdl1*	0.0000
Mitotic chromosome condensation	*Ncaph*, *Ncapg*, *Nusap1*	0.0058
Protein phosphorylation	*Ccnb1*, *Cdk1*, *Bub1*, *Bub1b*, *Ttk*, *Pbk*	0.0078
Mitotic cell cycle	*Ccnb1*, *Cdk1*, *Rrm2*	0.0378
Module 1: KEGG pathway		
Cell cycle	*Ccnb1*, *Cdk1*, *Bub1*, *Bub1b*, *Ttk*	0.0000
p53 signaling pathway	*Ccnb1*, *Cdk1*, *Rrm2*	0.0051
Progesterone-mediated oocyte maturation	*Ccnb1*, *Cdk1*, *Bub1*	0.0055
Oocyte meiosis	*Ccnb1*, *Cdk1*, *Bub1*	0.0064
Module 2: GO (BP)		
Chemokine-mediated signaling pathway	*Cxcl2*, *Cx3cr1*, *Ccl9*, *Ccl19*	0.0200
Positive regulation of ERK1 and ERK2 cascade	*Npy*, *C3*, *Ccl9*, *Ccl19*, *Gas6*	0.0161
Neuropeptide signaling pathway	*Npy*, *Oprk1*, *Npw*, *Npy1r*	0.0323
Module 2: KEGG pathway		
Chemokine signaling pathway	*Gnai1*, *Cxcl2*, *Cx3cr1*, *Ccl9*, *Ccl19*	0.0060
Module 3: GO (BP)		
Response to drug	*Lpl*, *CD36*, *Pparg*, *Pemt*, *Fabp4*, *Adipoq*	0.0000
Cellular response to insulin stimulus	*CD36*, *Pparg*, *Adipoq*, *Lpin1*	0.0034
Fatty acid oxidation	*CD36*, *Pparg*, *Adipoq*	0.0023
Brown fat cell differentiation	*Pparg*, *Fabp4*, *Adipoq*	0.0080
Fatty acid metabolic process	*CD36*, *Pparg*, *Fabp4*	0.0200
Module 3: KEGG pathway		
PPAR signaling pathway	*Lpl*, *CD36*, *Pparg*, *Fabp4*, *Adipoq*, *RGD1565355*	0.0000
Glycerophospholipid metabolism	*Cept1*, *Pemt*, *Lpcat3*, *Lpin1*	0.0016
AMPK signaling pathway	*CD36*, *Pparg*, *Adipoq*, *RGD1565355*	0.0025
Adipocytokine signaling pathway	*CD36*, *Adipoq*, *RGD1565355*	0.0174

GO (BP) and KEGG pathway enrichment analyses of the 3 modules demonstrated that the 3 most highly connected clusters in the PPI network were enriched in GO (BP) items or KEGG pathways including “cell division,” “cell cycle,” “protein phosphorylation,” “chemokine signaling pathway,” “neuropeptide signaling pathway,” “response to drug,” “cellular response to insulin stimulus,” “PPAR signaling pathway,” and “glycerophospholipid metabolism”.

**Table 4:** Hub DEGs/nodes of the PPI network.

Rank	Node	Connectivity degree	Regulation
Based on DEGs of module 1			
1	*Cdk1*	44	Up
2	*Bub1b*	32	Up
3	*Ccnb1*	32	Up
Based on the remaining DEGs of the PPI network			
1	*C3*	25	Up
2	*Agt*	17	Down
3	*Cxcl2*	17	Up
4	*Gnai1*	16	Down
5	*Mapk3*	15	Down
6	*Ccl9*	14	Up
7	*Cst3*	14	Down
8	*Cx3cr1*	14	Up
9	*Mmp9*	13	Up
10	*Npy*	12	Down

Hub DEGs of the PPI network were identified using the CytoHubba plugin in Cytoscape. A total of 13 DEGs (3 from module 1: *Cdk1*, *Bub1b*, and *Ccnb1*; 10 from the remaining DEGs: *C3*, *Agt*, *Cxcl2*, *Gnail*, *Mapk3*, *Ccl9*, *Cst3*, *Cx3cr1*, *Mmp9*, and *Npy*) with higher connectivity degree were identified as the overall hub DEGs of the whole PPI network.

**Table 5:** TF prediction of the hub DEGs.

TF	NES	No. of targets	No. of motifs
Nf1	7.519	7 (*C3*, *Cxcl2*, *Bub1b*, *Mapk3*, *Npy*, *Gnail*, *Agt*)	5
Kdm2a	7.270	3 (*C3*, *Npy*, *Gnail*)	3
Smad3	6.683	3 (*C3*, *Npy*, *Agt*)	2
Pbx3	6.324	3 (*Npy*, *Gnail*, *Agt*)	5
Rara	6.269	6 (*Cx3cr1*, *Bub1b*, *Mapk3*, *Npy*, *Gnail*, *Agt*)	9
Neurod1	6.069	5 (*Bub1b*, *Cst3*, *Npy*, *Gnail*, *Agt*)	3
Gfi1b	5.475	4 (*C3*, *Bub1b*, *Npy*, *Agt*)	2
Hoxa9	5.392	3 (*Cxcl2*, *Bub1b*, *Gnail*)	2
Esr2	5.392	3 (*C3*, *Gnail*, *Agt*)	4
Arid5a	5.364	7 (*Cxcl2*, *Bub1b*, *Cst3*, *Mapk3*, *Npy*, *Gnail*, *Agt*)	6
Plagl1	5.316	3 (*Bub1b*, *Npy*, *Agt*)	2

TF prediction of the hub DEGs was performed using the iRegulon plugin in Cytoscape with the criteria for motif enrichment analysis as follows: identity between orthologous genes ≥ 0.05, FDR on motif similarity ≤ 0.001, and normalized enrichment score (NES) > 5. Eleven TFs (Nf1, Kdm2a, Smad3, Pbx3, Rara, Neurod1, Gfi1b, Hoxa9, Esr2, Arid5a, and Plagl1) were predicted to regulate 9 of the 13 hub DEGs.

## Data Availability

No data were used to support this study.

## Supplementary Materials

Supplementary Materials Correlation plot of all samples and complete list of DEGs.
