# Supplementary material for: Gene Expression Profiling of the Sciatic Nerve in Streptozotocin-Induced Diabetic Rats with Peripheral Neuropathy
Source: J Diabetes Res. 2020 May 20;2020:5283284. doi: 10.1155/2020/5283284 (PMC7256683; doi:10.1155/2020/5283284)
Supplement: Supplementary Materials — Correlation plot of all samples and complete list of DEGs. [file 5283284.f1.pdf]

Supplemental Figure 1. Correlation plot of all samples

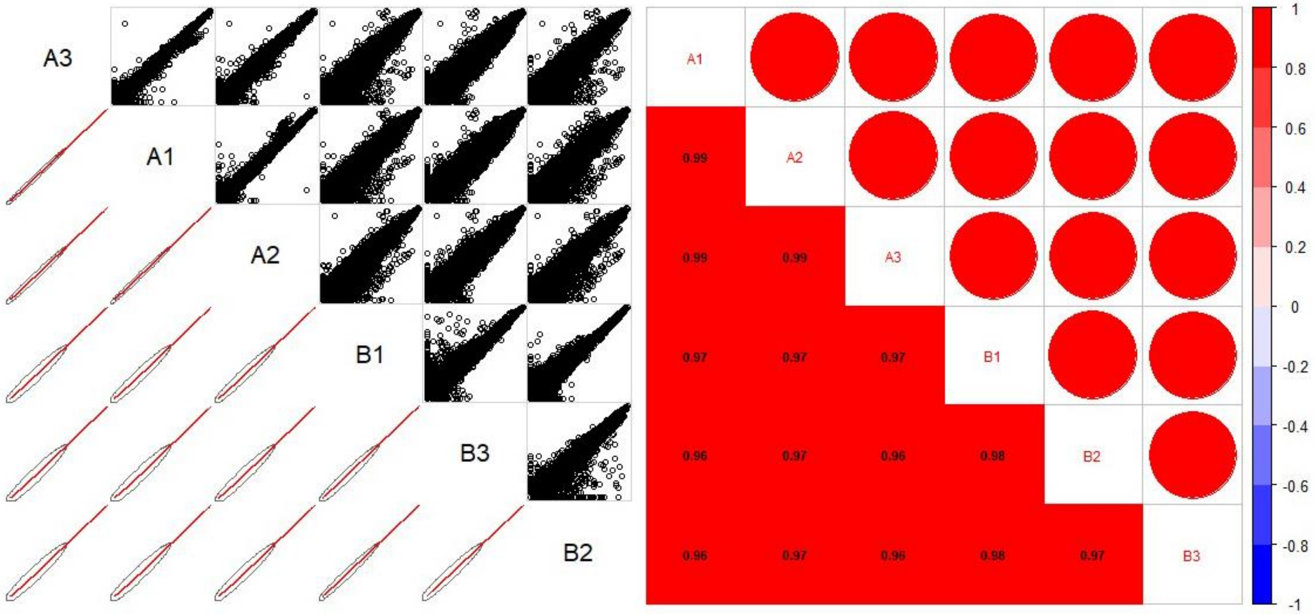

Pearson correlation analysis was used to assess the correlation between samples by using the R version 3.6.3. As shown, strong correlation was observed between samples within each group. Control group: A1, A2, A3; Experimental group: B1, B2, B3.

Supplemental Table 1. Complete list of DEGs

No	GeneSymbol	GeneName	Regulation	Fold Change	P-value	FDR
1	Ackr4	atypical chemokine receptor 4	up	5.0843429	0.000133522	0.007683641
2	Adamts7	ADAM metallopeptidase with thrombospondin type 1 motif, 7	up	2.1156522	0.000358794	0.011629805
3	Adcy6	adenylate cyclase 6	up	2.5215176	0.000882382	0.016346704
4	Adm	adrenomedullin	up	2.0199275	0.000304358	0.010906744
5	Aif1	allograft inflammatory factor 1	up	2.1460881	0.000771239	0.015531702
6	Ambp	alpha-1-microglobulin/bikunin precursor	up	7.6614017	9.78734E-05	0.007241839
7	Ankrd61	ankyrin repeat domain 61	up	2.8720675	0.000375442	0.01180017
8	Apoc1	apolipoprotein C-I	up	3.1743974	0.000821751	0.015845565
9	Arap3	ArfGAP with RhoGAP domain, ankyrin repeat and PH domain 3	up	2.0020368	0.000559187	0.013507393
10	Asgr1	asialoglycoprotein receptor 1	up	2.8621012	1.54444E-05	0.00466337
11	Aspm	asp (abnormal spindle) homolog, microcephaly associated (Drosophila)	up	7.6160818	1.62496E-05	0.004777529
12	Atp6v0d2	ATPase, H+ transporting, lysosomal V0 subunit D2	up	11.4131066	0.000149364	0.007978581
13	Bhlhe22	basic helix-loop-helix family, member e22	up	3.2377621	8.7433E-05	0.007046482
14	Btc	betacellulin	up	4.2482255	0.000735917	0.015215081
15	Bub1	BUB1 mitotic checkpoint serine/threonine kinase	up	2.1070516	2.82717E-05	0.005308839
16	Bub1b	BUB1 mitotic checkpoint serine/threonine kinase B	up	4.6507896	0.000254881	0.010173655
17	C3	complement component 3	up	18.6307292	0.000573282	0.013622858
18	Cabp2	calcium binding protein 2	up	8.7741555	0.000481153	0.012907801
19	Ccl3	chemokine (C-C motif) ligand 3	up	6.644307	0.000191162	0.009054145
20	Ccl9	chemokine (C-C motif) ligand 9	up	10.1685251	0.000201293	0.009222338
21	Ccnb1	cyclin B1	up	2.9271814	0.000355346	0.011612855
22	Ccnb2	cyclin B2	up	2.6724712	0.000905556	0.016544451

23	Ccnk	cyclin K	up	2.0464518	0.000232391	0.009783319
24	Cdh17	cadherin 17	up	3.1705883	0.000391608	0.011991874
25	Cdh6	cadherin 6	up	2.769434	0.000526479	0.013161429
26	Cdk1	cyclin-dependent kinase 1	up	5.5593146	0.000288002	0.010604886
27	Cenpw	centromere protein W	up	3.7302112	3.66704E-05	0.005674891
28	Ckap2	cytoskeleton associated protein 2	up	4.8059775	0.000100773	0.007241839
29	Cks2	CDC28 protein kinase regulatory subunit 2	up	6.4357779	3.50508E-06	0.002904359
30	Col22a1	collagen, type XXII, alpha 1	up	3.0485503	0.000424125	0.012307833
31	Cp	ceruloplasmin (ferroxidase)	up	2.8619064	0.000123686	0.007515143
32	Cramp1l	Crm, cramped-like (Drosophila)	up	5.4185666	0.000486288	0.0129488
33	Csrp2	cysteine and glycine-rich protein 2	up	2.9421219	0.000631849	0.014174351
34	Cthrc1	collagen triple helix repeat containing 1	up	11.6175805	0.000272348	0.010465194
35	Cuedc2	CUE domain containing 2	up	6.0334478	4.9224E-05	0.006448421
36	Cx3cr1	chemokine (C-X3-C motif) receptor 1	up	6.5982373	0.000194865	0.009138698
37	Cxcl2	chemokine (C-X-C motif) ligand 2	up	21.2002053	4.2039E-05	0.005935051
38	Cyfip2	cytoplasmic FMR1 interacting protein 2	up	3.5276141	0.000147337	0.007963063
39	Cym	chymosin	up	5.719453	6.62509E-05	0.00670157
40	Cyp11b3	cytochrome P450, family 11, subfamily b, polypeptide 3	up	2.6014446	0.000808511	0.015760882
41	Cyp2b2	cytochrome P450, family 2, subfamily b, polypeptide 2	up	2.0121323	0.000974396	0.01709426
42	Cyp3a9	cytochrome P450, family 3, subfamily a, polypeptide 9	up	4.576099	0.000710409	0.015063538
43	Cyp4a2	cytochrome P450, family 4, subfamily a, polypeptide 2	up	5.3994823	0.000559639	0.013507393
44	Cyp4f4	cytochrome P450, family 4, subfamily f, polypeptide 4	up	5.3582868	8.06873E-05	0.006840908
45	Def8	differentially expressed in FDCP 8 homolog (mouse)	up	2.8698254	0.000767397	0.015519914
46	Dio3	deiodinase, iodothyronine, type III	up	8.8632469	0.000187051	0.008984465

47	Dlk1	delta-like 1 homolog (Drosophila)	up	3.5013557	0.000498598	0.01302951
48	Dok3	docking protein 3	up	2.2211415	0.000268554	0.01043723
49	Dscc1	DNA replication and sister chromatid cohesion 1	up	2.2196389	0.00037032	0.011738559
50	Dsn1	DSN1, MIS12 kinetochore complex component	up	3.0346012	0.000721044	0.01509698
51	Dusp16	dual specificity phosphatase 16	up	3.2741944	8.6069E-05	0.007022284
52	Dusp6	dual specificity phosphatase 6	up	2.0393296	0.000628578	0.014171193
53	E2f8	E2F transcription factor 8	up	5.7601924	0.00047249	0.012820741
54	Eltd1	EGF, latrophilin and seven transmembrane domain containing 1	up	11.0187281	0.000247653	0.010023805
55	Epcam	epithelial cell adhesion molecule	up	8.6290815	0.000217356	0.009349496
56	Ephb3	Eph receptor B3	up	4.0929371	0.000208955	0.009348917
57	Extl3	exostosin-like glycosyltransferase 3	up	5.9688638	5.42632E-05	0.00647751
58	Fam131b	family with sequence similarity 131, member B	up	3.3731404	0.000799793	0.015687767
59	Fam19a5	family with sequence similarity 19, member A5	up	4.2108627	1.25434E-05	0.00466337
60	Fam72a	family with sequence similarity 72, member A	up	2.0514356	0.000259732	0.01024481
61	Fam78b	family with sequence similarity 78, member B	up	3.0026905	9.74215E-05	0.007241839
62	Folr4	folate receptor 4 (delta)	up	2.9642286	0.000284212	0.01057462
63	Foxm1	forkhead box M1	up	2.5603099	4.16682E-07	0.00192102
64	Fzd8	frizzled family receptor 8	up	2.3512382	0.000164753	0.008441015
65	Gamt	guanidinoacetate N-methyltransferase	up	2.2346708	8.26469E-05	0.006885665
66	Gins1	GINS complex subunit 1 (Psf1 homolog)	up	2.6688607	0.00022338	0.009537291
67	Glra2	glycine receptor, alpha 2	up	4.6188595	3.64476E-05	0.005674891
68	Golm1	golgi membrane protein 1	up	3.8256786	0.000759299	0.015454568
69	Gpr68	G protein-coupled receptor 68	up	2.0378888	0.000853308	0.01612149
70	Grk4	G protein-coupled receptor kinase 4	up	2.4655822	4.18497E-06	0.002904676

71	Gsg1	germ cell associated 1	up	2.6719996	0.000207974	0.00934862
72	Hmgb2	high mobility group box 2	up	2.763117	0.000152817	0.008090446
73	Homer1	homer homolog 1 (Drosophila)	up	2.8834054	5.34955E-05	0.00647751
74	Hoxc5	homeo box C5	up	4.6054597	0.000610372	0.014090613
75	Iglon5	IgLON family member 5	up	5.1081263	0.000428427	0.012356966
76	Igsf8	immunoglobulin superfamily, member 8	up	2.1705039	0.000393181	0.01200442
77	Il1rn	interleukin 1 receptor antagonist	up	4.8148083	0.000144874	0.007937271
78	Ipo13	importin 13	up	2.7855597	0.000237111	0.00979265
79	Itih2	inter-alpha-trypsin inhibitor heavy chain 2	up	4.6421937	0.000785083	0.015628271
80	Kcng3	potassium voltage-gated channel, subfamily G, member 3	up	2.801903	0.000496807	0.01302951
81	Kif20a	kinesin family member 20A	up	5.4021504	1.0661E-05	0.004553205
82	Kif20b	kinesin family member 20B	up	4.8143714	2.8679E-05	0.005308839
83	Kif22	kinesin family member 22	up	6.1414604	5.02188E-05	0.006448421
84	Klhl2	kelch-like family member 2	up	2.0789572	1.43548E-05	0.00466337
85	Knstrn	kinetochore-localized astrin/SPAG5 binding protein	up	5.9635933	0.000117922	0.00746415
86	Kntc1	kinetochore associated 1	up	4.1022398	0.000933476	0.016822342
87	LOC100362981	LRRGT00010-like	up	3.9284281	2.04639E-05	0.004994303
88	LOC171161	common salivary protein 1	up	7.7198212	2.24082E-05	0.005124869
89	LOC303448	similar to glyceraldehyde-3-phosphate dehydrogenase	up	2.3689804	0.000546005	0.013361708
90	LOC307263	similar to Glyceraldehyde-3-phosphate dehydrogenase (GAPDH)	up	5.2732949	3.18943E-05	0.005527659
91	LOC681185	similar to centromere autoantigen H	up	3.9442994	0.000395041	0.01200442
92	LOC681325	hypothetical protein LOC681325	up	2.3717164	0.000764525	0.015499517
93	LOC681382	hypothetical protein LOC681382	up	4.1164255	7.81293E-05	0.006768556
94	LOC689665	hypothetical protein LOC689665	up	8.4802665	0.000791743	0.015666516

95	LOC689757	similar to osteoclast inhibitory lectin	up	2.2015169	0.000503528	0.013067581
96	LOC690309	similar to DNA methyltransferase 3B	up	7.2510031	0.000724815	0.015111056
97	Lppr1	lipid phosphate phosphatase-related protein type 1	up	3.0442411	0.000571176	0.013607704
98	Lrrc8e	leucine rich repeat containing 8 family, member E	up	3.7458071	0.000163017	0.008370077
99	Mapk12	mitogen-activated protein kinase 12	up	3.8669466	0.000277657	0.010480355
100	Med12	mediator complex subunit 12	up	2.5821963	5.36619E-05	0.00647751
101	Melk	maternal embryonic leucine zipper kinase	up	7.427877	1.99638E-06	0.002503247
102	Mixl1	Mix paired-like homeobox 1	up	3.6391435	3.40053E-05	0.005625991
103	Mki67	marker of proliferation Ki-67	up	9.0458679	8.27691E-06	0.004159438
104	Mmp12	matrix metallopeptidase 12	up	90.6725927	7.21572E-05	0.00670157
105	Mmp9	matrix metallopeptidase 9	up	5.3004768	0.000198658	0.009154403
106	Mrap2	melanocortin 2 receptor accessory protein 2	up	10.8959068	0.000179661	0.008825267
107	Msln	mesothelin	up	3.9256922	0.000439777	0.012450484
108	Mtfp1	mitochondrial fission process 1	up	6.683634	0.000770779	0.015531702
109	Mycn	v-myc avian myelocytomatosis viral oncogene neuroblastoma derived homolog	up	3.3282792	0.000150455	0.008000998
110	Ncapg	non-SMC condensin I complex, subunit G	up	4.9967316	0.000207499	0.009345869
111	Ncaph	non-SMC condensin I complex, subunit H	up	4.4697916	0.000115624	0.00746415
112	Ndc80	NDC80 kinetochore complex component	up	4.588126	2.75138E-06	0.002731207
113	Nefm	neurofilament, medium polypeptide	up	9.7980705	0.000969696	0.017088539
114	Neurog2	neurogenin 2	up	8.2784295	0.000373937	0.01176839
115	Ninl	ninein-like	up	2.119698	0.000225128	0.009594723
116	Notch1	notch 1	up	12.4914611	0.000331851	0.011364368
117	Nppc	natriuretic peptide C	up	2.0062557	0.000789655	0.015651207

118	Nuf2	NUF2, NDC80 kinetochore complex component	up	4.6083268	1.70471E-05	0.004893139
119	Nusap1	nucleolar and spindle associated protein 1	up	3.4560773	3.99852E-05	0.005738602
120	Olfml2a	olfactomedin-like 2A	up	2.3137464	2.77853E-05	0.005295663
121	Olr379	olfactory receptor 379	up	5.6012766	0.000591342	0.013879927
122	Olr619	olfactory receptor 619	up	5.3811119	0.000497818	0.01302951
123	Olr804	olfactory receptor 804	up	3.4904984	5.83951E-06	0.003567194
124	Oprk1	opioid receptor, kappa 1	up	2.6530312	0.000822075	0.015845565
125	Pbk	PDZ binding kinase	up	6.6070648	9.64838E-05	0.007241839
126	Pcdh17	protocadherin 17	up	3.5083167	1.4274E-06	0.002503247
127	Pcdhb15	protocadherin beta 15	up	2.2080359	0.000960251	0.017021594
128	Pi4k2a	phosphatidylinositol 4-kinase type 2 alpha	up	2.0932176	0.000466981	0.012785364
129	Plxnb3	plexin B3	up	2.9649637	8.05094E-05	0.006840908
130	Prc1	protein regulator of cytokinesis 1	up	3.6954728	0.000103091	0.007271432
131	Ptk7	protein tyrosine kinase 7	up	2.0039808	0.000319263	0.011150098
132	Ptprn2	protein tyrosine phosphatase, receptor type, N polypeptide 2	up	4.0807005	5.08739E-05	0.006452293
133	Ptprv	protein tyrosine phosphatase, receptor type, V	up	2.1845885	0.000617502	0.014122807
134	Pura	purine rich element binding protein A	up	2.7676228	0.000215041	0.009348917
135	Pwp2	PWP2 periodic tryptophan protein homolog (yeast)	up	6.8175104	0.000787321	0.015628271
136	Rad51	RAD51 recombinase	up	2.8660761	0.000991888	0.017261307
137	Rassf2	Ras association (RalGDS/AF-6) domain family member 2	up	5.050669	0.000124362	0.007515143
138	Rbp1	retinol binding protein 1, cellular	up	3.3019425	7.07089E-05	0.00670157
139	Rdh10	retinol dehydrogenase 10 (all-trans)	up	2.057703	0.000240294	0.009853295
140	Reg3b	regenerating islet-derived 3 beta	up	2.4178831	0.000912953	0.016573841
141	Rfc3	replication factor C (activator 1) 3	up	2.2992473	3.43127E-05	0.005634447

142	RGD1306926	similar to hypothetical protein FLJ22175	up	3.0230665	0.000329631	0.011332084
143	RGD1561327	similar to melanoma antigen family A, 10	up	2.7137372	0.000211868	0.009348917
144	Rgs1	regulator of G-protein signaling 1	up	8.2964179	0.000497387	0.01302951
145	Rgs12	regulator of G-protein signaling 12	up	2.4261231	9.76605E-05	0.007241839
146	Ripk3	receptor-interacting serine-threonine kinase 3	up	2.7737126	0.00023544	0.00979265
147	Rrm2	ribonucleotide reductase M2	up	3.848191	0.000357538	0.011629805
148	RT1-M4	RT1 class Ib, locus M4	up	7.330075	0.000885294	0.016375196
149	Rwdd2a	RWD domain containing 2A	up	2.1602024	0.000422461	0.012307833
150	Sapcd1	suppressor APC domain containing 1	up	2.3701891	0.000189974	0.009029968
151	Sapcd2	suppressor APC domain containing 2	up	3.7907091	0.000908677	0.016557331
152	Sbsn	suprabasin	up	6.2000161	0.000111644	0.00746415
153	Scn3b	sodium channel, voltage-gated, type III, beta	up	11.9309623	0.000797386	0.015687767
154	Sh3gl3	SH3-domain GRB2-like 3	up	3.1572519	0.000635147	0.014202512
155	Slc39a6	solute carrier family 39 (zinc transporter), member 6	up	2.2307111	1.50542E-05	0.00466337
156	Slfn13	schlafen family member 13	up	3.3079642	4.58764E-05	0.006161164
157	Smarcc1	SWI/SNF related, matrix associated, actin dependent regulator of chromatin, subfamily c, member 1	up	2.0727017	0.000691235	0.014822656
158	Smyd5	SMYD family member 5	up	4.3140036	0.000136051	0.007760843
159	Socs5	suppressor of cytokine signaling 5	up	2.067298	9.20766E-05	0.007210707
160	Sox4	SRY (sex determining region Y)-box 4	up	4.7676566	0.000291858	0.010687427
161	Sox5	SRY (sex determining region Y)-box 5	up	3.1288113	0.000144926	0.007937271
162	Spag5	sperm associated antigen 5	up	2.7608631	2.3255E-07	0.001846755
163	Spc24	SPC24, NDC80 kinetochore complex component	up	2.8607297	1.37855E-05	0.00466337
164	Spdl1	spindle apparatus coiled-coil protein 1	up	2.3366765	8.00894E-05	0.006840408

165	Spdya	speedy/RINGO cell cycle regulator family member A	up	2.5987002	5.00965E-05	0.006448421
166	Spic	Spi-C transcription factor (Spi-1/PU.1 related)	up	2.2296186	0.000136623	0.007763771
167	Stambp	Stam binding protein	up	3.8261515	0.000333784	0.011364368
168	Strc	stereocilin	up	2.0027939	0.00096608	0.017086781
169	Tagln3	transgelin 3	up	12.7660108	0.000496414	0.01302951
170	Tead2	TEA domain family member 2	up	3.08482	0.000507362	0.013089751
171	Tk1	thymidine kinase 1, soluble	up	5.0644253	1.2181E-05	0.00466337
172	Tmem202	transmembrane protein 202	up	3.4645481	0.000563886	0.013543191
173	Tmem47	transmembrane protein 47	up	5.2241573	7.24388E-05	0.00670157
174	Tmem88	transmembrane protein 88	up	2.0456826	5.79082E-05	0.006550663
175	Tmsbl1	thymosin beta-like protein 1	up	5.505763	0.000147402	0.007963063
176	Tnfrsf12a	tumor necrosis factor receptor superfamily, member 12a	up	5.6286666	1.35508E-05	0.00466337
177	Tnn	tenascin N	up	2.5117894	0.000857636	0.016154409
178	Tnnt1	troponin T type 1 (skeletal, slow)	up	2.9308066	0.000325813	0.011258322
179	Tspan11	tetraspanin 11	up	2.7235789	0.000154638	0.008118338
180	Ttk	Ttk protein kinase	up	4.3313116	0.000767163	0.015519914
181	Ube2o	ubiquitin-conjugating enzyme E2O	up	7.2795592	0.000455653	0.012691886
182	Ube2t	ubiquitin-conjugating enzyme E2T (putative)	up	8.722121	0.000471054	0.012820741
183	Ucn2	urocortin 2	up	44.2013083	0.000198004	0.009154403
184	Vwa5b1	von Willebrand factor A domain containing 5B1	up	2.2513069	0.00047088	0.012820741
185	Vwce	von Willebrand factor C and EGF domains	up	4.8589755	0.000980531	0.017163973
186	Wdr38	WD repeat domain 38	up	3.2674311	0.000564489	0.013543191
1	Aacs	acetoacetyl-CoA synthetase	down	4.5079324	0.000325895	0.011258322
2	Aadat	aminoadipate aminotransferase	down	4.2378257	0.000525645	0.013154365

3	Abcd2	ATP-binding cassette, subfamily D (ALD), member 2	down	5.9946387	0.00015959	0.00828339
4	Abhd5	abhydrolase domain containing 5	down	3.7859046	0.000118092	0.00746415
5	Abhd6	abhydrolase domain containing 6	down	2.0092335	0.000590984	0.013879927
6	Acat2	acetyl-CoA acetyltransferase 2	down	2.4509414	0.000524864	0.013154365
7	Acsl1	acyl-CoA synthetase long-chain family member 1	down	5.7869279	2.25844E-06	0.002562152
8	Acss2	acyl-CoA synthetase short-chain family member 2	down	2.6406979	1.82485E-06	0.002503247
9	Acta2	actin, alpha 2, smooth muscle, aorta	down	2.2345668	4.99052E-05	0.006448421
10	Acvr1c	activin A receptor, type IC	down	6.2263773	0.000701051	0.014965812
11	Adamtsl3	ADAMTS-like 3	down	4.6504659	0.00064012	0.014249485
12	Adarb1	adenosine deaminase, RNA-specific, B1	down	2.3401957	8.54878E-05	0.007016519
13	Adipoq	adiponectin, C1Q and collagen domain containing	down	3.4768898	0.000710058	0.015063538
14	Adk	adenosine kinase	down	2.1079441	0.000334474	0.011364368
15	Adrb3	adrenoceptor beta 3	down	4.6306452	6.591E-05	0.00670157
16	Agmo	alkylglycerol monooxygenase	down	4.9929099	0.000388129	0.011915947
17	Agpat2	1-acylglycerol-3-phosphate O-acyltransferase 2	down	2.8790953	3.8661E-05	0.005720871
18	Agpat3	1-acylglycerol-3-phosphate O-acyltransferase 3	down	3.1323223	0.000118893	0.00746415
19	Agpat9	1-acylglycerol-3-phosphate O-acyltransferase 9	down	3.7168219	9.22716E-05	0.007210707
20	Agt	angiotensinogen (serpin peptidase inhibitor, clade A, member 8)	down	4.3480961	9.3221E-05	0.007210707
21	Ahrr	aryl-hydrocarbon receptor repressor	down	3.139942	0.000315982	0.011106777
22	Ajuba	ajuba LIM protein	down	2.1511577	0.000123843	0.007515143
23	Akap12	A kinase (PRKA) anchor protein 12	down	3.2712621	0.000583503	0.013777382
24	Akr1b1	aldo-keto reductase family 1, member B1 (aldose reductase)	down	4.9428302	0.000130497	0.00762445
25	Akr1c14	aldo-keto reductase family 1, member C14	down	3.2829364	0.000440509	0.012450484
26	Alg6	ALG6, alpha-1,3-glucosyltransferase	down	4.8679819	0.000336716	0.011378599

27	Amigo2	adhesion molecule with Ig like domain 2	down	2.7881303	1.34812E-05	0.00466337
28	Aoc3	amine oxidase, copper containing 3	down	3.0906087	0.000113526	0.00746415
29	Aplp2	amyloid beta (A4) precursor-like protein 2	down	2.9400489	0.00021086	0.009348917
30	Ar	androgen receptor	down	3.0314791	0.00025418	0.010173655
31	Armc1	armadillo repeat containing 1	down	2.0487496	0.000500979	0.01302951
32	Atp2a3	ATPase, Ca++ transporting, ubiquitous	down	3.1882218	0.000326887	0.01127026
33	Atp6v0e1	ATPase, H+ transporting, lysosomal, V0 subunit e1	down	2.1282842	0.000118331	0.00746415
34	Atp9b	ATPase, class II, type 9B	down	2.1752241	0.000306956	0.010914799
35	Bche	butyrylcholinesterase	down	2.4679456	0.000284584	0.01057462
36	Cacna1c	calcium channel, voltage-dependent, L type, alpha 1C subunit	down	3.0449591	0.000488535	0.012975321
37	Cacnb3	calcium channel, voltage-dependent, beta 3 subunit	down	3.4019965	0.000116025	0.00746415
38	Cald1	caldesmon 1	down	2.2039913	9.68986E-05	0.007241839
39	Caprin1	cell cycle associated protein 1	down	2.0651949	0.000706712	0.015032767
40	Car13	carbonic anhydrase 13	down	3.5644971	3.05673E-06	0.002800908
41	Car3	carbonic anhydrase 3	down	6.992138	0.000355874	0.011614159
42	Cat	catalase	down	3.1842059	0.000339751	0.01143253
43	Cav1	caveolin 1, caveolae protein	down	2.8508497	0.000956709	0.016985085
44	Cav2	caveolin 2	down	5.9095448	3.38001E-06	0.002904359
45	Ccl19	chemokine (C-C motif) ligand 19	down	2.7296334	0.00062188	0.014137089
46	Ccng2	cyclin G2	down	2.3102468	0.000243552	0.009919136
47	Cd302	CD302 molecule	down	2.2416971	7.48632E-05	0.006705041
48	Cd36	CD36 molecule (thrombospondin receptor)	down	4.5947849	0.000715198	0.015078647
49	Cd55	Cd55 molecule	down	4.3231207	0.000177224	0.008777922
50	Cd99l2	CD99 molecule-like 2	down	4.4345478	2.27469E-05	0.005124869

51	Cdc40	cell division cycle 40	down	2.5910433	0.000738274	0.015215081
52	Cdc42bpa	CDC42 binding protein kinase alpha	down	2.2791779	2.92882E-05	0.005367406
53	Cdc42ep3	CDC42 effector protein (Rho GTPase binding) 3	down	2.3318341	0.000373709	0.01176839
54	Cdkl1	cyclin-dependent kinase-like 1 (CDC2-related kinase)	down	2.5199517	0.00096803	0.017088539
55	Cdo1	cysteine dioxygenase type 1	down	8.0907288	1.24249E-05	0.00466337
56	Cdr2	cerebellar degeneration-related 2	down	2.1875856	0.000420985	0.012307833
57	Cenpb	centromere protein B	down	2.2369702	0.000629096	0.014171193
58	Cep70	centrosomal protein 70	down	2.4593807	4.24458E-05	0.00594841
59	Cept1	choline/ethanolamine phosphotransferase 1	down	2.7884456	0.00097115	0.017088539
60	Cers2	ceramide synthase 2	down	3.3979905	0.00092421	0.01670591
61	Ces1d	carboxylesterase 1D	down	4.1051976	0.000384092	0.011867124
62	Cgref1	cell growth regulator with EF hand domain 1	down	2.6732036	7.34848E-05	0.00670157
63	Chchd2	coiled-coil-helix-coiled-coil-helix domain containing 2	down	2.3240866	0.000785561	0.015628271
64	Chga	chromogranin A (parathyroid secretory protein 1)	down	5.2419388	0.000635273	0.014202512
65	Chmp4c	charged multivesicular body protein 4C	down	2.3382006	0.00021113	0.009348917
66	Chn1	chimerin 1	down	3.5916589	0.000334373	0.011364368
67	Cldn12	claudin 12	down	2.4733632	9.61919E-06	0.004353374
68	Cldn14	claudin 14	down	2.2218254	7.42325E-05	0.00670157
69	Cldn5	claudin 5	down	3.7617483	0.000864597	0.016211264
70	Cmtm6	CKLF-like MARVEL transmembrane domain containing 6	down	2.6116909	2.87458E-05	0.005308839
71	Cntf	ciliary neurotrophic factor	down	3.6039558	1.36393E-05	0.00466337
72	Cobll1	cordon-bleu WH2 repeat protein-like 1	down	2.4479159	0.000128741	0.007610736
73	Cog6	component of oligomeric golgi complex 6	down	2.1853229	7.29912E-05	0.00670157
74	Col24a1	collagen, type XXIV, alpha 1	down	6.6325725	0.00069896	0.014947965

75	Col4a1	collagen, type IV, alpha 1	down	3.1234357	0.000235169	0.00979265
76	Col4a2	collagen, type IV, alpha 2	down	2.7662683	7.34346E-05	0.00670157
77	Col4a5	collagen, type IV, alpha 5	down	2.9703576	0.000214259	0.009348917
78	Col9a2	collagen, type IX, alpha 2	down	7.7365951	0.000258332	0.010223424
79	Crebl2	cAMP responsive element binding protein-like 2	down	2.3361661	0.00015426	0.008118338
80	Cryab	crystallin, alpha B	down	2.7874572	1.74283E-06	0.002503247
81	Csgalnact2	chondroitin sulfate N-acetylgalactosaminyltransferase 2	down	5.826693	2.50604E-06	0.002595819
82	Cst3	cystatin C	down	2.2852944	0.000257265	0.010215136
83	Ctdspl	CTD (carboxy-terminal domain, RNA polymerase II, polypeptide A) small phosphatase-like	down	2.8313403	0.000219077	0.009387194
84	Ctnna2	catenin (cadherin associated protein), alpha 2	down	6.4777149	9.28149E-05	0.007210707
85	Cyb561	cytochrome b-561	down	4.0860244	3.53536E-06	0.002904359
86	Cyp39a1	cytochrome P450, family 39, subfamily a, polypeptide 1	down	2.2467358	0.000311499	0.011015257
87	Cyp51	cytochrome P450, family 51	down	2.2527658	0.00068599	0.014776698
88	Cyyr1	cysteine/tyrosine-rich 1	down	2.3382772	5.64437E-07	0.00192102
89	Dbh	dopamine beta-hydroxylase (dopamine beta-monooxygenase)	down	8.0642036	2.25099E-06	0.002562152
90	Dbnl	drebrin-like	down	2.78943	0.000342639	0.011446095
91	Ddah1	dimethylarginine dimethylaminohydrolase 1	down	4.7323431	0.000265678	0.010359283
92	Dgat2	diacylglycerol O-acyltransferase 2	down	3.8871236	0.000630518	0.014171193
93	Dgkb	diacylglycerol kinase, beta	down	2.0743906	0.0009416	0.016913293
94	Dll1	delta-like 1 (Drosophila)	down	4.6526438	2.07537E-05	0.004994303
95	Dmbt1	deleted in malignant brain tumors 1	down	3.2916657	0.000669674	0.014582397
96	Drp2	dystrophin related protein 2	down	4.7527368	0.000160601	0.008299697
97	Dusp14	dual specificity phosphatase 14	down	3.4144961	6.75401E-06	0.003742034

98	Ebf3	early B-cell factor 3	down	2.9759244	0.000306924	0.010914799
99	Echdc1	enoyl CoA hydratase domain containing 1	down	2.5609402	0.00071513	0.015078647
100	Echs1	enoyl CoA hydratase, short chain, 1, mitochondrial	down	2.1662173	0.000233249	0.009783319
101	Efhd1	EF-hand domain family, member D1	down	7.2909201	0.000607736	0.014056996
102	Egfl7	EGF-like-domain, multiple 7	down	2.04654	6.20272E-05	0.006693911
103	Ehbp1	EH domain binding protein 1	down	2.0267157	0.000369281	0.011738559
104	Elmo2	engulfment and cell motility 2	down	2.6728583	2.26346E-05	0.005124869
105	Elmod2	ELMO/CED-12 domain containing 2	down	2.7164718	0.000394304	0.01200442
106	Elovl6	ELOVL fatty acid elongase 6	down	5.3907836	0.000182594	0.008844594
107	Enc1	ectodermal-neural cortex 1	down	3.1111558	0.000721848	0.01509698
108	Enoph1	enolase-phosphatase 1	down	2.1002082	0.000865544	0.016211264
109	Enpep	glutamyl aminopeptidase	down	2.8945597	0.00018848	0.009017198
110	Eps8	epidermal growth factor receptor pathway substrate 8	down	2.1546934	0.000521993	0.013154365
111	Eps8l2	EPS8-like 2	down	2.4949014	0.000797951	0.015687767
112	Ermn	ermin, ERM-like protein	down	2.3853019	0.000927216	0.016734836
113	Esam	endothelial cell adhesion molecule	down	2.8264815	6.70455E-05	0.00670157
114	Etnk1	ethanolamine kinase 1	down	2.1770765	0.000103228	0.007271432
115	Fabp4	fatty acid binding protein 4, adipocyte	down	4.1613934	0.000624494	0.014139906
116	Faim3	Fas apoptotic inhibitory molecule 3	down	3.4809598	0.000104183	0.007278725
117	Fam196a	family with sequence similarity 196, member A	down	2.7148278	0.000481802	0.012907801
118	Fam213a	family with sequence similarity 213, member A	down	2.2551022	3.46546E-05	0.005634447
119	Fam45a	family with sequence similarity 45, member A	down	2.0209999	5.32758E-07	0.00192102
120	Fam46c	family with sequence similarity 46, member C	down	3.271465	0.000903818	0.016538073
121	Fam57a	family with sequence similarity 57, member A	down	2.7581177	0.00013774	0.007776097

122	Fam57b	family with sequence similarity 57, member B	down	6.3423426	0.000269986	0.010442987
123	Fam81a	family with sequence similarity 81, member A	down	2.1586819	0.000849204	0.016082223
124	Fat4	FAT atypical cadherin 4	down	2.5511379	1.80112E-06	0.002503247
125	Fcgrt	Fc fragment of IgG, receptor, transporter, alpha	down	2.2342542	5.78506E-05	0.006550663
126	Ffar4	free fatty acid receptor 4	down	6.8249818	0.00065771	0.014455049
127	Fgfr1	Fibroblast growth factor receptor 1	down	3.0815823	0.000515294	0.013147662
128	Fitm2	fat storage-inducing transmembrane protein 2	down	2.4297496	0.000199948	0.009196053
129	Fmo2	flavin containing monooxygenase 2	down	2.4678584	8.75486E-05	0.007046482
130	Fpgt	fucose-1-phosphate guanylyltransferase	down	2.3548039	0.000284839	0.01057462
131	Frmpd1	FERM and PDZ domain containing 1	down	2.8886819	0.000567221	0.013554147
132	Frrs1l	ferric-chelate reductase 1-like	down	6.6114608	0.000253881	0.010173655
133	Fzd4	frizzled family receptor 4	down	2.9846288	0.000215474	0.009348917
134	Gabarapl2	GABA(A) receptor-associated protein like 2	down	2.0231522	0.000615365	0.014110167
135	Galnt16	UDP-N-acetyl-alpha-D-galactosamine:polypeptide N-acetylgalactosaminyltransferase 16	down	3.4577023	1.56663E-05	0.004665417
136	Galntl5	UDP-N-acetyl-alpha-D-galactosamine:polypeptide N-acetylgalactosaminyltransferase-like 5	down	5.553432	0.000364099	0.011643364
137	Gas6	growth arrest specific 6	down	2.121799	0.000744456	0.01528934
138	Gatsl3	GATS protein-like 3	down	2.1574511	0.000919459	0.016657936
139	Gca	grancalcin	down	2.9106218	0.000126724	0.007554442
140	Gcc2	GRIP and coiled-coil domain containing 2	down	2.164161	7.71426E-05	0.006755632
141	Gcnt2	glucosaminyl (N-acetyl) transferase 2, I-branching enzyme	down	2.1144911	0.00021094	0.009348917
142	Gdf10	growth differentiation factor 10	down	3.5879381	0.000268992	0.01043723
143	Ghr	growth hormone receptor	down	4.4632427	9.30775E-05	0.007210707

144	Gla	galactosidase, alpha	down	2.3500786	7.31061E-06	0.003786262
145	Glod4	glyoxalase domain containing 4	down	2.9307838	0.000119399	0.00746415
146	Gls2	glutaminase 2 (liver, mitochondrial)	down	2.0276253	0.000556018	0.013475653
147	Gm2a	GM2 ganglioside activator	down	2.0022663	0.000841395	0.016051692
148	Gmcl1	germ cell-less, spermatogenesis associated 1	down	2.1882356	0.000520818	0.013154365
149	Gnai1	guanine nucleotide binding protein (G protein), alpha inhibiting activity polypeptide 1	down	3.7879397	2.72122E-05	0.005228254
150	Gpr116	G protein-coupled receptor 116	down	2.2030563	0.000621468	0.014137089
151	Gpt	glutamic-pyruvate transaminase (alanine aminotransferase)	down	2.9950449	0.000950037	0.016985085
152	Grem2	gremlin 2	down	2.4842635	0.000271332	0.010442987
153	Gria2	glutamate receptor, ionotropic, AMPA 2	down	4.6738	0.000331732	0.011364368
154	Gucy1b3	guanylate cyclase 1, soluble, beta 3	down	2.4699686	0.000384546	0.011867124
155	Hapln2	hyaluronan and proteoglycan link protein 2	down	8.857771	9.86745E-06	0.004353374
156	Has2	hyaluronan synthase 2	down	3.3982566	0.000418571	0.012307833
157	Hbegf	heparin-binding EGF-like growth factor	down	2.5436773	0.000147221	0.007963063
158	Hhex	hematopoietically expressed homeobox	down	2.0584924	0.000172892	0.008653337
159	Hiat1	hippocampus abundant transcript 1	down	2.7837696	0.000877689	0.016310498
160	Hip1r	huntingtin interacting protein 1 related	down	2.0189817	0.000760081	0.015454568
161	Hist1h4b	histone cluster 1, H4b	down	7.3161289	5.94793E-05	0.006583067
162	Hmgcr	3-hydroxy-3-methylglutaryl-CoA reductase	down	3.0812548	0.000676746	0.014683787
163	Hrh1	histamine receptor H 1	down	2.7487421	0.000214483	0.009348917
164	Hsd17b12	hydroxysteroid (17-beta) dehydrogenase 12	down	3.2899296	3.81121E-05	0.005674891
165	Hsd17b7	hydroxysteroid (17-beta) dehydrogenase 7	down	2.4218012	0.000973579	0.01709426
166	Igfbp5	insulin-like growth factor binding protein 5	down	3.2679627	1.37412E-05	0.00466337

167	Igflr1	IGF-like family receptor 1	down	5.3763569	0.000116267	0.00746415
168	Il33	interleukin 33	down	3.6105756	0.000857653	0.016154409
169	Inadl	InaD-like (Drosophila)	down	2.6241655	0.000233222	0.009783319
170	Ino80c	INO80 complex subunit C	down	2.0889448	0.000142132	0.007889814
171	Insig1	insulin induced gene 1	down	5.5983639	0.000115835	0.00746415
172	Iqgap2	IQ motif containing GTPase activating protein 2	down	2.9231401	5.47098E-05	0.00647751
173	Itpr2	inositol 1,4,5-trisphosphate receptor, type 2	down	2.1369208	0.000520947	0.013154365
174	Jag2	jagged 2	down	3.5969336	0.000883529	0.016355244
175	Jam2	junctional adhesion molecule 2	down	2.8680119	0.000198096	0.009154403
176	Katnbl1	katanin p80 subunit B-like 1	down	3.2938436	0.000198251	0.009154403
177	Kcnb1	potassium voltage gated channel, Shab-related subfamily, member 1	down	3.7075667	3.64072E-05	0.005674891
178	Kcnh2	potassium voltage-gated channel, subfamily H (eag-related), member 2	down	4.9984231	0.000210425	0.009348917
179	Kcnk3	potassium channel, subfamily K, member 3	down	2.8690219	0.000820481	0.015845565
180	Kdr	kinase insert domain receptor	down	2.2199697	0.000410136	0.012231263
181	Klf11	Kruppel-like factor 11	down	2.0712732	0.000225611	0.009597571
182	Klf15	Kruppel-like factor 15	down	2.0299877	0.00036301	0.011643364
183	Kras	Kirsten rat sarcoma viral oncogene	down	2.0725971	0.000625668	0.014139906
184	Lifr	leukemia inhibitory factor receptor alpha	down	3.5235862	0.000847773	0.016082223
185	Limch1	LIM and calponin homology domains 1	down	3.2108463	3.93535E-05	0.005738602
186	Lims2	LIM and senescent cell antigen like domains 2	down	2.6410422	2.04531E-05	0.004994303
187	Llgl1	lethal giant larvae homolog 1 (Drosophila)	down	2.038973	0.000857928	0.016154409
188	LOC100365047	scavenger receptor class B, member 2-like	down	6.6524886	0.000198589	0.009154403
189	LOC100909539	uncharacterized LOC100909539	down	3.4735229	0.0007453	0.01528934
190	LOC100910088	prolow-density lipoprotein receptor-related protein 1-like	down	2.7783043	0.000456021	0.012691886

191	LOC100910823	derlin-2-like	down	2.4253514	0.000721996	0.01509698
192	LOC100912608	homeobox protein Hox-A10-like	down	2.285261	0.000840843	0.016051692
193	LOC291863	carboxylesterase-like	down	7.1389434	0.000852301	0.016115253
194	LOC365829	similar to grancalcin	down	3.3771688	9.78131E-05	0.007241839
195	LOC501445	derlin-1-like	down	3.4863331	0.000442564	0.012477699
196	LOC680225	similar to Tubulin alpha-3 chain (Alpha-tubulin 3)	down	2.1119419	0.000431123	0.012397184
197	LOC685067	similar to guanylate binding protein family, member 6	down	2.1116671	1.07026E-05	0.004553205
198	LOC686911	similar to Exocyst complex component 1	down	2.3740386	6.52817E-05	0.00670157
199	LOC688613	hypothetical protein LOC688613	down	3.4652434	5.88416E-05	0.006550663
200	Lox	lysyl oxidase	down	2.2614769	0.000757917	0.015454568
201	Lpcat3	lysophosphatidylcholine acyltransferase 3	down	2.4323158	0.000254939	0.010173655
202	Lpin1	lipin 1	down	2.5613416	0.000894909	0.016463564
203	Lpl	lipoprotein lipase	down	8.3815477	0.000347273	0.011474934
204	Lrch4	leucine-rich repeats and calponin homology (CH) domain containing 4	down	2.7995407	0.00061536	0.014110167
205	Lrg1	leucine-rich alpha-2-glycoprotein 1	down	2.223327	0.000253097	0.010173655
206	Lrp11	low density lipoprotein receptor-related protein 11	down	2.0000975	5.38123E-06	0.003373751
207	Lrp2	low density lipoprotein receptor-related protein 2	down	7.8052204	8.77131E-06	0.004264648
208	Lrrc16a	leucine rich repeat containing 16A	down	2.2828985	0.000250276	0.010106045
209	Lrrc58	leucine rich repeat containing 58	down	3.4741329	0.000626159	0.014139906
210	Lrrn1	leucine rich repeat neuronal 1	down	10.9943385	0.000478479	0.012870073
211	Ly6c	Ly6-C antigen	down	2.0637592	0.000291533	0.010687427
212	Lypd6b	LY6/PLAUR domain containing 6B	down	7.2419201	9.07579E-05	0.007183442
213	Man2a1	mannosidase, alpha, class 2A, member 1	down	2.3304228	0.000870125	0.016255494
214	Maoa	monoamine oxidase A	down	2.5457537	4.60329E-05	0.006161164

215	Maob	monoamine oxidase B	down	5.3023682	0.000278348	0.010480355
216	Mapk3	mitogen activated protein kinase 3	down	2.111467	0.000454563	0.012680917
217	Mapk8ip1	mitogen-activated protein kinase 8 interacting protein 1	down	3.1646033	0.000886981	0.016391746
218	March2	membrane-associated ring finger (C3HC4) 2, E3 ubiquitin protein ligase	down	3.6965174	7.05014E-05	0.00670157
219	Mboat1	membrane bound O-acyltransferase domain containing 1	down	2.3034642	1.45107E-05	0.00466337
220	Mecom	MDS1 and EVI1 complex locus	down	3.2757524	0.00065561	0.014435531
221	Mgll	monoglyceride lipase	down	3.5609236	0.000712668	0.01506438
222	Mgst1	microsomal glutathione S-transferase 1	down	3.1394995	0.000548493	0.013380302
223	Mif4gd	MIF4G domain containing	down	2.1939538	3.7359E-05	0.005674891
224	Mmd	monocyte to macrophage differentiation-associated	down	4.7705926	0.000188489	0.009017198
225	Mmp24	matrix metallopeptidase 24	down	4.1656452	0.000527309	0.013168364
226	Mrc1	mannose receptor, C type 1	down	3.5097414	0.000501149	0.01302951
227	Mrvi1	murine retrovirus integration site 1 homolog	down	2.1086827	0.000316551	0.011106777
228	Msmo1	methylsterol monooxygenase 1	down	2.6805834	0.000874568	0.016303373
229	Mst1	Macrophage stimulating 1 (hepatocyte growth factor-like)	down	3.4892164	0.000784783	0.015628271
230	Mt3	metallothionein 3	down	2.2809392	0.000273018	0.010474031
231	Mtch2	mitochondrial carrier 2	down	2.1036861	0.000602968	0.01403545
232	Mturn	maturin, neural progenitor differentiation regulator homolog (Xenopus)	down	2.0280743	0.000287754	0.010604886
233	Mvd	mevalonate (diphospho) decarboxylase	down	2.254388	0.000178464	0.008802744
234	Mylk	myosin light chain kinase	down	2.9046423	7.18634E-05	0.00670157
235	Nadk2	NAD kinase 2, mitochondrial	down	2.0009683	2.29627E-05	0.005124869
236	Nat8l	N-acetyltransferase 8-like	down	3.0989039	0.000762577	0.015488185
237	Nbl1	neuroblastoma 1, DAN family BMP antagonist	down	2.5106186	0.000939252	0.016900866
238	Ncoa4	nuclear receptor coactivator 4	down	3.6306034	0.000653645	0.014435531

239	Ndfip2	Nedd4 family interacting protein 2	down	2.0658035	0.00079573	0.015687767
240	Nedd4	neural precursor cell expressed, developmentally down-regulated 4, E3 ubiquitin protein ligase	down	2.1301829	1.28305E-05	0.00466337
241	Nipa1	non imprinted in Prader-Willi/Angelman syndrome 1	down	2.0777083	0.000294444	0.010713224
242	Nnmt	nicotinamide N-methyltransferase	down	2.7230346	8.57038E-05	0.007016519
243	Nova1	neuro-oncological ventral antigen 1	down	3.0778463	0.000295891	0.010713224
244	Npw	neuropeptide W	down	6.1824097	4.73863E-06	0.003051168
245	Npy	neuropeptide Y	down	2.5047038	0.000842963	0.01605335
246	Npy1r	neuropeptide Y receptor Y1	down	2.3876922	0.000238389	0.00979265
247	Nqo1	NAD(P)H dehydrogenase, quinone 1	down	3.948015	9.43093E-06	0.004353374
248	Nrtn	neurturin	down	3.0813823	0.000103102	0.007271432
249	Nucb1	nucleobindin 1	down	3.0817321	0.000720386	0.01509698
250	Nxt2	nuclear transport factor 2-like export factor 2	down	3.9430402	6.35E-05	0.006693911
251	Osbpl11	oxysterol binding protein-like 11	down	2.0697794	0.000343998	0.011446095
252	Pard6b	par-6 family cell polarity regulator beta	down	2.5545109	4.08004E-05	0.005820536
253	Pc	pyruvate carboxylase	down	3.1814825	0.000530352	0.013202816
254	Pcsk6	proprotein convertase subtilisin/kexin type 6	down	4.3273926	5.13731E-05	0.006475725
255	Pdcd4	programmed cell death 4	down	2.9007655	2.45172E-05	0.005124869
256	Pde2a	phosphodiesterase 2A, cGMP-stimulated	down	3.1347586	2.50622E-05	0.005140955
257	Pemt	phosphatidylethanolamine N-methyltransferase	down	2.6677568	0.000257132	0.010215136
258	Pex11a	peroxisomal biogenesis factor 11 alpha	down	2.6229101	1.90948E-06	0.002503247
259	Pex19	peroxisomal biogenesis factor 19	down	2.124333	3.7763E-05	0.005674891
260	Pgf	placental growth factor	down	2.5178227	0.000215995	0.009348917
261	Phf20l1	PHD finger protein 20-like 1	down	2.8578715	0.000996291	0.01727485

262	Phgdh	phosphoglycerate dehydrogenase	down	3.2935216	0.000226098	0.009597571
263	Pi15	peptidase inhibitor 15	down	2.3328284	9.61577E-05	0.007241839
264	Pip4k2a	phosphatidylinositol-5-phosphate 4-kinase, type II, alpha	down	2.3036736	0.000103181	0.007271432
265	Pipox	pipecolic acid oxidase	down	3.0332661	0.00080676	0.015741394
266	Pkhd1l1	polycystic kidney and hepatic disease 1-like 1	down	4.3111149	9.11914E-05	0.007193855
267	Pla2g15	phospholipase A2, group XV	down	2.2653896	0.000546101	0.013361708
268	Plcb1	phospholipase C, beta 1 (phosphoinositide-specific)	down	2.8352822	6.66001E-05	0.00670157
269	Plcl1	phospholipase C-like 1	down	2.8925133	6.9823E-06	0.003780598
270	Plscr4	phospholipid scramblase 4	down	4.612056	0.000108162	0.007404739
271	Plvap	plasmalemma vesicle associated protein	down	3.8728107	0.00061945	0.014122807
272	Pmp2	peripheral myelin protein 2	down	6.8035466	1.87771E-05	0.004929144
273	Pofut1	protein O-fucosyltransferase 1	down	2.1526441	0.000200791	0.009217036
274	Pparg	peroxisome proliferator-activated receptor gamma	down	4.2267135	0.000294954	0.010713224
275	Ppp2r2b	protein phosphatase 2, regulatory subunit B, beta	down	3.6572261	5.28531E-05	0.00647751
276	Ppp4r4	protein phosphatase 4, regulatory subunit 4	down	3.9903024	0.000286292	0.01057462
277	Prkar2b	protein kinase, cAMP dependent regulatory, type II beta	down	6.1213195	1.90836E-05	0.004941824
278	Prkch	protein kinase C, eta	down	2.3214588	7.56544E-05	0.006740076
279	Prkci	protein kinase C, iota	down	2.0716424	1.64438E-05	0.004777529
280	Procr	protein C receptor, endothelial	down	2.9396317	0.000730237	0.015167533
281	Prph	peripherin	down	2.128174	0.00073603	0.015215081
282	Psat1	phosphoserine aminotransferase 1	down	4.1437692	1.78909E-05	0.004899215
283	Psmf1	proteasome (prosome, macropain) inhibitor subunit 1	down	2.2712319	9.9053E-05	0.007241839
284	Ptgds	prostaglandin D2 synthase (brain)	down	4.1464456	0.000489616	0.012989551
285	Ptprd	protein tyrosine phosphatase, receptor type, D	down	4.6572753	0.00034625	0.011457033

286	Ptprr	protein tyrosine phosphatase, receptor type, R	down	2.5333877	0.000529939	0.013202816
287	Pttg1ip	pituitary tumor-transforming 1 interacting protein	down	2.5692789	0.000434005	0.012397184
288	Pxdn	peroxidasin homolog (Drosophila)	down	2.0244229	0.000911126	0.016557331
289	Rab11a	RAB11a, member RAS oncogene family	down	2.6502045	0.000787675	0.015628271
290	Rab6b	RAB6B, member RAS oncogene family	down	2.1900958	0.000845648	0.016082223
291	Rapgef5	Rap guanine nucleotide exchange factor (GEF) 5	down	3.7676258	2.28667E-05	0.005124869
292	Rarres1	retinoic acid receptor responder (tazarotene induced) 1	down	2.8669088	0.00048645	0.0129488
293	Rasip1	Ras interacting protein 1	down	2.5473364	3.89483E-06	0.002904676
294	Rb1	retinoblastoma 1	down	2.2228337	0.000703263	0.01498617
295	Rbm12b	RNA binding motif protein 12B	down	4.3193742	6.08933E-05	0.006654692
296	Rbp4	retinol binding protein 4, plasma	down	4.522627	6.06003E-06	0.003609351
297	Rbpj	recombination signal binding protein for immunoglobulin kappa J region	down	2.027186	0.000243565	0.009919136
298	Rbpms	RNA binding protein with multiple splicing	down	2.6120377	1.77957E-05	0.004899215
299	Rcan2	regulator of calcineurin 2	down	2.6414234	5.56442E-05	0.006513124
300	Rcbtb2	regulator of chromosome condensation (RCC1) and BTB (POZ) domain containing protein 2	down	2.0179984	0.000951241	0.016985085
301	Rell2	RELT-like 2	down	2.6422109	0.00018181	0.008844594
302	Ret	ret proto-oncogene	down	5.0647737	1.76277E-05	0.004899215
303	Rgcc	regulator of cell cycle	down	2.0079282	0.000736085	0.015215081
304	RGD1309594	similar to RIKEN cDNA 1810043G02; DNA segment, Chr 10, Johns Hopkins University 13, expressed	down	4.7496246	6.29302E-05	0.006693911
305	RGD1309651	similar to 1190005I06Rik protein	down	3.454667	0.000305974	0.010914799
306	RGD1310110	similar to 3632451O06Rik protein	down	3.5641404	0.000553866	0.013475653
307	RGD1310769	similar to HSPC288	down	5.1461911	0.000689886	0.014807062

308	RGD1359108	similar to RIKEN cDNA 3110043O21	down	3.5515622	5.96853E-05	0.006583067
309	RGD1562284	similar to Glutaminyl-peptide cyclotransferase precursor (QC)	down	3.3732233	9.51084E-05	0.007241839
310	RGD1564149	similar to Protein C21orf58	down	4.1207381	0.000516183	0.013147662
311	RGD1565355	similar to fatty acid translocase/CD36	down	4.4375524	0.000901223	0.016528669
312	RGD621098	similar to RIKEN cDNA D230025D16Rik	down	2.9594033	4.21014E-05	0.005935051
313	Rhbdl1	rhomboid, veinlet-like 1 (Drosophila)	down	2.4123206	0.000119845	0.00746415
314	Rhobtb3	Rho-related BTB domain containing 3	down	3.3776734	0.000993114	0.017270029
315	Rhof	ras homolog family member F (in filopodia)	down	2.4232421	7.26988E-05	0.00670157
316	Rin2	Ras and Rab interactor 2	down	4.6707183	0.000343516	0.011446095
317	Ripk2	receptor-interacting serine-threonine kinase 2	down	2.9862573	0.000332521	0.011364368
318	Rmdn2	regulator of microtubule dynamics 2	down	2.9291945	0.000351604	0.011545168
319	Rnf181	ring finger protein 181	down	2.3989005	3.86714E-06	0.002904676
320	Robo3	roundabout, axon guidance receptor, homolog 3 (Drosophila)	down	4.4239379	8.38033E-06	0.004159438
321	Rph3al	rabphilin 3A-like (without C2 domains)	down	2.1853174	0.000472079	0.012820741
322	Rrn3	RRN3 RNA polymerase I transcription factor homolog (S. cerevisiae)	down	2.7202536	0.000518223	0.013148196
323	S100b	S100 calcium binding protein B	down	3.9797956	8.36022E-05	0.006939854
324	S1pr1	sphingosine-1-phosphate receptor 1	down	5.0849843	0.000238398	0.00979265
325	Sc5d	sterol-C5-desaturase	down	3.8791578	2.86834E-05	0.005308839
326	Scnn1b	sodium channel, non-voltage-gated 1, beta subunit	down	2.4324703	0.000893332	0.016447241
327	Sdccag3	serologically defined colon cancer antigen 3	down	2.0086947	0.000175629	0.008729824
328	Selenbp1	selenium binding protein 1	down	2.1171444	0.000956076	0.016985085
329	Sepp1	selenoprotein P, plasma, 1	down	2.8079385	0.000130573	0.00762445
330	Sept8	septin 8	down	2.5461481	0.000406053	0.012153038
331	Serf2	small EDRK-rich factor 2	down	2.9250354	8.01072E-05	0.006840408

332	Serinc1	serine incorporator 1	down	3.148404	0.000235089	0.00979265
333	Serp1	stress-associated endoplasmic reticulum protein 1	down	2.2029662	1.501E-06	0.002503247
334	Sft2d2	SFT2 domain containing 2	down	2.8696937	0.000289852	0.010656526
335	Sh3gl2	SH3-domain GRB2-like 2	down	2.5669153	5.48909E-05	0.00647751
336	Shank3	SH3 and multiple ankyrin repeat domains 3	down	2.3314411	0.000259352	0.01024481
337	She	Src homology 2 domain containing E	down	2.133307	0.000742615	0.015278109
338	Shox2	short stature homeobox 2	down	3.4830808	8.44753E-05	0.006963808
339	Siah1a	seven in absentia 1A	down	2.135433	0.000119032	0.00746415
340	Slc15a2	solute carrier family 15 (oligopeptide transporter), member 2	down	3.9821679	3.36248E-05	0.005625991
341	Slc1a3	solute carrier family 1 (glial high affinity glutamate transporter), member 3	down	3.2367419	1.47068E-05	0.00466337
342	Slc22a3	solute carrier family 22 (organic cation transporter), member 3	down	6.0665529	2.65099E-05	0.005176812
343	Slc25a16	solute carrier family 25 (mitochondrial carrier, Graves disease autoantigen), member 16	down	2.1511617	0.000120045	0.00746415
344	Slc25a36	solute carrier family 25 (pyrimidine nucleotide carrier), member 36	down	2.8078883	0.000433897	0.012397184
345	Slc35a5	solute carrier family 35, member A5	down	3.2520912	0.000919046	0.016657936
346	Slc38a10	solute carrier family 38, member 10	down	2.3431605	0.000472097	0.012820741
347	Slc40a1	solute carrier family 40 (iron-regulated transporter), member 1	down	2.0534358	0.000657	0.014452796
348	Slc43a3	solute carrier family 43, member 3	down	2.3079984	0.000311778	0.011015257
349	Slc45a3	solute carrier family 45, member 3	down	2.7183073	1.25993E-05	0.00466337
350	Slc7a10	solute carrier family 7 (neutral amino acid transporter light chain, asc system), member 10	down	6.1193984	0.00066235	0.014503515
351	Slco2b1	solute carrier organic anion transporter family, member 2b1	down	2.061843	5.60439E-05	0.006513124
352	Slfn5	schlafen family member 5	down	3.1321323	6.19282E-05	0.006693911

353	Smpd3	sphingomyelin phosphodiesterase 3, neutral membrane	down	2.3931808	0.000284294	0.01057462
354	Snx13	sorting nexin 13	down	3.0781673	0.000522549	0.013154365
355	Sod3	superoxide dismutase 3, extracellular	down	2.453857	0.000117406	0.00746415
356	Sorl1	sortilin-related receptor, LDLR class A repeats-containing	down	2.5368282	0.000492632	0.01302605
357	Spns2	spinster homolog 2	down	2.6177751	6.79111E-05	0.00670157
358	Sppl2a	signal peptide peptidase-like 2A	down	2.2566306	0.000605168	0.014056996
359	Srd5a1	steroid-5-alpha-reductase, alpha polypeptide 1 (3-oxo-5 alpha-steroid delta 4-dehydrogenase alpha 1)	down	2.8698387	0.000509149	0.013099323
360	Srgn	serglycin	down	2.4009422	5.22676E-05	0.00647751
361	St13	suppression of tumorigenicity 13	down	3.0279544	0.000561296	0.013507393
362	Stard3nl	STARD3 N-terminal like	down	2.4802536	1.48588E-05	0.00466337
363	Stard7	StAR-related lipid transfer (START) domain containing 7	down	2.4946374	3.02462E-05	0.005434408
364	Stc1	stanniocalcin 1	down	2.9573458	0.000266533	0.01037564
365	Stx12	syntaxin 12	down	2.2796518	0.000500631	0.01302951
366	Stx3	syntaxin 3	down	3.0087872	0.000325386	0.011258322
367	Sulf1	sulfatase 1	down	2.5106693	0.000263988	0.010359283
368	Sumf1	sulfatase modifying factor 1	down	2.2969307	0.000809102	0.015760882
369	Syngr3	synaptogyrin 3	down	3.8491408	4.25916E-07	0.00192102
370	Tbc1d15	TBC1 domain family, member 15	down	2.0256557	0.000379714	0.011867124
371	Tc2n	tandem C2 domains, nuclear	down	3.4606625	0.000265508	0.010359283
372	Tcf25	transcription factor 25 (basic helix-loop-helix)	down	2.2569288	5.77224E-05	0.006550663
373	Thbd	thrombomodulin	down	2.0362848	0.000317293	0.01111645
374	Thrsp	thyroid hormone responsive	down	5.1551366	0.00057318	0.013622858
375	Timp4	tissue inhibitor of metalloproteinase 4	down	2.5512764	2.72054E-05	0.005228254

376	Tm7sf2	transmembrane 7 superfamily member 2	down	5.2754929	0.000258203	0.010223424
377	Tmbim6	transmembrane BAX inhibitor motif containing 6	down	2.7704993	0.000943639	0.016918707
378	Tmcc3	transmembrane and coiled-coil domain family 3	down	2.7603106	5.35629E-05	0.00647751
379	Tmeff1	transmembrane protein with EGF-like and two follistatin-like domains 1	down	4.415078	0.000648663	0.01437559
380	Tmem252	transmembrane protein 252	down	4.0388828	0.000655031	0.014435531
381	Tmem62	transmembrane protein 62	down	2.1360022	0.000217804	0.009349496
382	Tnfrsf11b	tumor necrosis factor receptor superfamily, member 11b	down	3.4964382	0.0001246	0.007515143
383	Tpd52	tumor protein D52	down	2.1091201	0.000740066	0.01523884
384	Trem3	triggering receptor expressed on myeloid cells 3	down	2.9600792	0.000477186	0.012870073
385	Trex1	three prime repair exonuclease 1	down	3.1594151	0.000827889	0.015931846
386	Trhr	thyrotropin releasing hormone receptor	down	3.8746623	0.000813862	0.015794361
387	Tril	TLR4 interactor with leucine-rich repeats	down	2.4704941	2.51208E-05	0.005140955
388	Tspan12	tetraspanin 12	down	6.6039438	0.000654478	0.014435531
389	Tspan17	tetraspanin 17	down	3.4101856	0.000821602	0.015845565
390	Tspan2	tetraspanin 2	down	2.3741367	0.000346075	0.011457033
391	Tspyl4	TSPY-like 4	down	3.6236474	0.000622574	0.014139364
392	Ttc23	tetratricopeptide repeat domain 23	down	2.031059	0.000976642	0.017121066
393	Tuba1c	tubulin, alpha 1C	down	2.3785662	0.000108109	0.007404739
394	Ube2e2	ubiquitin-conjugating enzyme E2E 2	down	2.413441	0.00068969	0.014807062
395	Ube2v2	ubiquitin-conjugating enzyme E2 variant 2	down	2.2578665	0.000760273	0.015454568
396	Ucma	upper zone of growth plate and cartilage matrix associated	down	3.2666891	0.000546269	0.013361708
397	Vkorc1l1	vitamin K epoxide reductase complex, subunit 1-like 1	down	2.0487149	0.000810405	0.015760882
398	Vsig2	V-set and immunoglobulin domain containing 2	down	2.6882446	0.000517154	0.013147662
399	Vsnl1	visinin-like 1	down	5.0782438	0.000958761	0.017007828

400	Wdr44	WD repeat domain 44	down	2.2129666	0.000555941	0.013475653
401	Wnt2	wingless-type MMTV integration site family member 2	down	4.127601	0.000142526	0.007889814
402	Wnt5a	wingless-type MMTV integration site family, member 5A	down	3.3011188	0.000312093	0.011015257
403	Yy1	YY1 transcription factor	down	2.1073888	0.000270835	0.010442987
404	Zdhhc17	zinc finger, DHHC-type containing 17	down	2.5811649	2.95433E-05	0.005372829
405	Zdhhc3	zinc finger, DHHC-type containing 3	down	2.0524669	0.000606836	0.014056996
406	Zfp180	zinc finger protein 180	down	2.7313888	0.000149031	0.007978581
407	Zfp36l2	zinc finger protein 36, C3H type-like 2	down	2.1735963	0.000663741	0.014511282
408	Zfp521	zinc finger protein 521	down	2.0607179	7.16962E-05	0.00670157
409	Znrf2	zinc and ring finger 2	down	2.1162948	0.000112318	0.00746415
410	Zwint	ZW10 interacting kinetochore protein	down	2.1723317	1.18392E-05	0.00466337
411	Zzef1	zinc finger, ZZ-type with EF hand domain 1	down	2.0480021	7.17666E-05	0.00670157
